# Using the Job Dispatcher Web Interfaces for Sequence Analysis

**DOI:** 10.1002/cpz1.70416

**Published:** 2026-09-26

**Authors:** Nandana Madhusoodanan, Fábio Madeira, Ania Niewielska, Tariq Sulaiman Fazil Ahamed, Leeroy M. Ncube, Timothy Porter

**Affiliations:** ^1^ European Molecular Biology Laboratory‐European Bioinformatics Institute (EMBL‐EBI) Wellcome Trust Genome Campus Hinxton Cambridge United Kingdom

**Keywords:** bioinformatics tools, Job Dispatcher, sequence alignment, sequence analysis, sequence search, workflows

## Abstract

The rapid growth of biological sequence data has increased the need for easy, accessible, and scalable tools for sequence comparison and analysis. The EMBL‐EBI Job Dispatcher framework helps meet this need by providing a single web‐based platform that offers many commonly used tools, including the BLAST, Clustal Omega, and EMBOSS programs. In this article, we provide a practical, easy‐to‐follow guide to the main tool categories available in Job Dispatcher, focusing on multiple sequence alignment and sequence similarity search. We present step‐by‐step workflows that demonstrate how to run these analyses directly on the web without installing any bioinformatics software. Our goal is to provide a simple, practical reference for researchers and students new to bioinformatics, helping them choose the right tools and use web‐based analysis effectively. © 2026 EMBL‐EBI. *Current Protocols* published by Wiley Periodicals LLC.

**Basic Protocol 1**: Sequence similarity search using FASTA

**Basic Protocol 2**: Sequence similarity search using NCBI BLAST+

**Basic Protocol 3**: Sequence similarity search using PSI‐BLAST

**Basic Protocol 4**: Multiple sequence alignment using Clustal Omega

**Basic Protocol 5**: Visualizing multiple sequence alignments using MView

**Basic Protocol 6**: Workflow from BLAST results to Clustal Omega to MView

## INTRODUCTION

For decades, the European Bioinformatics Institute (EMBL‐EBI) has provided access to an extensive collection of biological databases and analysis tools that support research worldwide, and it continues to do so (Thakur et al., 2026). The Job Dispatcher service provides and maintains a comprehensive range of freely available bioinformatics analysis tools through both web interfaces and programmatic web services (Madeira, Madhusoodanan, Lee, Eusebi, Niewielska, Tivey, Lopez, et al., 2024; see Current Protocols article: Madeira, Madhusoodanan, Lee, Eusebi, Niewielska, Tivey, Meacham, et al., 2024). Over time, these services have been continually enhanced for greater usability, integration, and performance. The EMBL‐EBI Job Dispatcher framework now offers a unified, browser‐based system for executing sequence analysis tasks. Currently, the platform includes over 50 tools across 10 categories, covering tools ranging from sequence similarity search (SSS) to protein functional analysis.

Through the Job Dispatcher website, users can run analyses directly online without local installations or command‐line expertise. Job Dispatcher (https://www.ebi.ac.uk/jdispatcher/) tools are grouped and organized into several categories, such as Sequence Similarity Searching (https://www.ebi.ac.uk/jdispatcher/sss), Multiple Sequence Alignment (https://www.ebi.ac.uk/jdispatcher/msa), Pairwise Sequence Alignment (https://www.ebi.ac.uk/jdispatcher/psa), and Protein Functional Analysis (https://www.ebi.ac.uk/jdispatcher/pfa), each with dedicated pages. These pages explain the purpose of each category, detail the capabilities of individual tools, and offer guidance on selecting the most suitable tool based on input data and analysis objectives. The organized layout enables users to quickly identify the appropriate tools for their research questions.

The Job Dispatcher website provides access to widely used applications such as NCBI BLAST+ (Altschul et al., 1997; Camacho et al., 2009; also see Current Protocols article: Ladunga, 2002) for SSSs, Clustal Omega (Sievers et al., 2011; Sievers et al., 2014; Sievers & Higgins, 2018) for multiple sequence alignment (MSA), and the EMBOSS suite (Rice et al., 2000) for sequence analysis. These tools accept both protein and nucleotide input sequence data, and a few offer workflows that pass results directly to subsequent tools. Table 1 lists the analysis tools, their corresponding categories, and the URLs of their web interfaces.

**Table 1:** EMBL‐EBI Bioinformatics Sequence Analysis Tools and Tool Categories

Tool category	Tools included	Main URL
Sequence Similarity Searching	NCBI BLAST+, FASTA, FASTM/S/F, PSI‐BLAST, PSI‐Search, SSEARCH, GGSEARCH, GLSEARCH, PSI‐Search2, HMMER3 phmmer, HMMER 3 nhmmer	https://www.ebi.ac.uk/jdispatcher/sss/
Multiple Sequence Alignment	Clustal Omega, Kalign, MAFFT, MUSCLE, T‐Coffee, WebPRANK, MView	https://www.ebi.ac.uk/jdispatcher/psa/
Protein Functional Analysis	InterProScan 5, Phobius, Pratt, RADAR, HMMER 3 hmmscan, PfamScan	https://www.ebi.ac.uk/jdispatcher/pfa/
Sequence Format Conversion	Seqret, MView	https://www.ebi.ac.uk/jdispatcher/sfc/
Phylogeny Analysis	Simple Phylogeny	https://www.ebi.ac.uk/jdispatcher/phylogeny/
Pairwise Sequence Alignment	Needle, Stretcher, Water, Matcher, LALIGN, GeneWise, GGSEARCH2SEQ, SSEARCH2SEQ	https://www.ebi.ac.uk/jdispatcher/psa/
RNA Analysis	Infernal cmscan, MapMi, R2DT	https://www.ebi.ac.uk/jdispatcher/rna/
Sequence Operation	SeqCksum	https://www.ebi.ac.uk/jdispatcher/so/
Sequence Translation	Transeq, Sixpack, Backtranseq, Backtransmbig	https://www.ebi.ac.uk/jdispatcher/st/
Sequence Statistics	Pepinfo, Pepstats, Pepwindow, SAPS, Cpgplot, Newcpgreport, Isochore, Dotmatcher, Dotpath, Dottup, Polydot	https://www.ebi.ac.uk/jdispatcher/seqstats/
EMBOSS Tools	Needle, Stretcher, Water, Matcher, Transeq, Sixpack, Backtranseq, Backtransmbig, Pepinfo, Pepstats, Pepwindow, Cpgplot, Newcpgreport, Isochore, Seqret, Dotmatcher, Dotpath, Dottup, Polydot	https://www.ebi.ac.uk/jdispatcher/emboss/

In the following sections, we outline strategic considerations for using the Job Dispatcher web interface and provide detailed, step‐by‐step procedures for using key tools across these categories.

Basic Protocols 1 and 2 describe how to perform SSS using FASTA and NCBI BLAST+, respectively. We then explore how to perform iterative searches using PSI‐BLAST (Basic Protocol 3), which is a useful tool to find distant evolutionary relationships and remote homogs among protein sequences. Basic Protocol 4 describes how to generate MSAs using Clustal Omega, which are then visualized using MView (Basic Protocol 5). Finally, Basic Protocol 6 provides a complete workflow, starting from a sequence search using NCBI BLAST+ (as described in Basic Protocol 2), taking the hit sequences and generating MSAs with Clustal Omega (as described in Basic Protocol 4), and then visualizing with MView (as described in Basic Protocol 5).

## STRATEGIC PLANNING

### Benefits of Use

The decision to use the EMBL‐EBI Job Dispatcher web interface for sequence analysis involves balancing accessibility, flexibility, and computational needs. The web interface provides direct, browser‐based access to a comprehensive range of bioinformatics tools and databases, allowing researchers to perform analyses without installing software or maintaining local compute infrastructure.

The Job Dispatcher web interface is particularly valuable in the following contexts:

**Ease of access**: Users can run analyses from any modern web browser without needing local installations or scripting experience.

**Integration of tools**: It supports workflows that link multiple applications, facilitating end‐to‐end online sequence analysis.

**Maintenance‐free operation**: Software and databases are maintained and updated by EMBL‐EBI teams, ensuring users consistently access the latest versions of tools and datasets.

**Visualization and output handling**: The interface provides result visualization options, including alignment views and summary reports, reducing the need for external visualization software.

### Considerations and Limitations

Although the web interface offers significant advantages, there are certain situations where it may not be optimal:

**Large‐scale or automated analyses**: For high‐throughput or systematic analysis, programmatic access via Web Services or local installations may be more appropriate (see Current Protocols article: Madeira, Madhusoodanan, Lee, Eusebi, Niewielska, Tivey, Meacham, et al., 2024).

**Job size and runtime constraints**: Web‐based submissions are typically subject to input size limits and are not scalable, which may restrict extensive analyses.

### Planning for Effective Use

For most users, especially those conducting small‐ to medium‐scale sequence analyses or exploring bioinformatics workflows for educational or research purposes, the Job Dispatcher web interface provides an accessible and practical solution. It enables interactive exploration of sequence data and promotes reproducible, standardized approaches to analysis through consistent access to tools and integrated visualization features. Researchers planning regular‐ or large‐scale analyses may benefit from combining web‐based analyses with programmatic access or local installations to gain greater flexibility.

## Basic Protocol 1 SEQUENCE SIMILARITY SEARCH USING FASTA

SSS is a computational method that identifies related DNA, RNA, or protein sequences by aligning a query sequence against a database of known sequences. By statistically assessing how well database and query sequences match, one can infer homology and transfer information to the query sequence. The Job Dispatcher SSS category provides analysis tools from the following suites: NCBI BLAST+ (Altschul et al., 1997; Camacho et al., 2009; also see Current Protocols article: Ladunga, 2002), FASTA (Pearson & Lipman, 1988; also see Current Protocols article: Pearson, 2016), and HMMER3 (Finn et al., 2015). Iterative sequence search tools are also provided, namely PSI‐BLAST (Altschul et al., 1997; Camacho et al., 2009; also see Current Protocols article: Ladunga, 2002), PSI‐Search, and PSI‐Search 2 (Li et al., 2012).

The FASTA tool suite provides a comprehensive set of similarity/homology‐searching programs (FASTA, SSEARCH, GGSEARCH, GLSEARCH, FASTX, FASTY, FASTM, FASTF, and FASTS), which are similar to those offered by NCBI BLAST+, as well as additional programs for searching with short peptides and oligonucleotides.

### Necessary Resources

#### Hardware

Any computer with internet access.

##### Software

A modern web browser (e.g., Chrome, Firefox, Edge, Safari).

##### Input

A nucleotide or protein sequence in a supported format (e.g., Fasta, EMBL, GenBank, PHYLIP, UniProtKB/Swiss‐Prot) can be pasted into the form or uploaded.
Alternatively, a database identifier in the format database:identifier can be used (e.g., UniProtKB:GSTM1_MOUSE).

##### Example for this protocol

The mouse protein Glutathione S‐transferase Mu 1 (UniProtKB:P10649) will be used as input:
  Entry details: https://www.uniprot.org/uniprotkb/P10649/entry
  Fasta‐format sequence: https://rest.uniprot.org/uniprotkb/P10649.fasta

**Figure 1:**
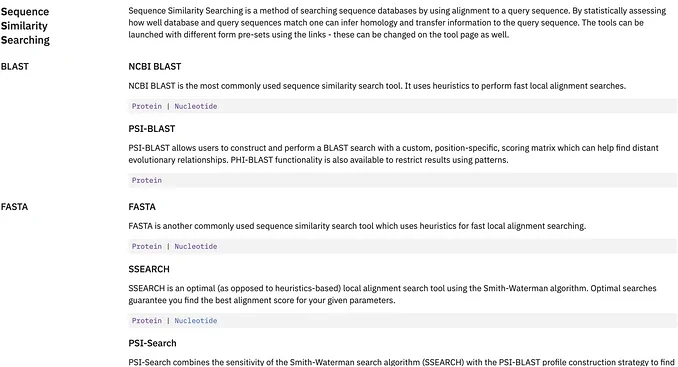
Job Dispatcher Sequence Similarity Search tools category page.

1 Open a web browser and navigate to the SSS page https://www.ebi.ac.uk/jdispatcher/sss (Fig. 1).
  This page lists available tools for protein and nucleotide SSS.

**Figure 2:**
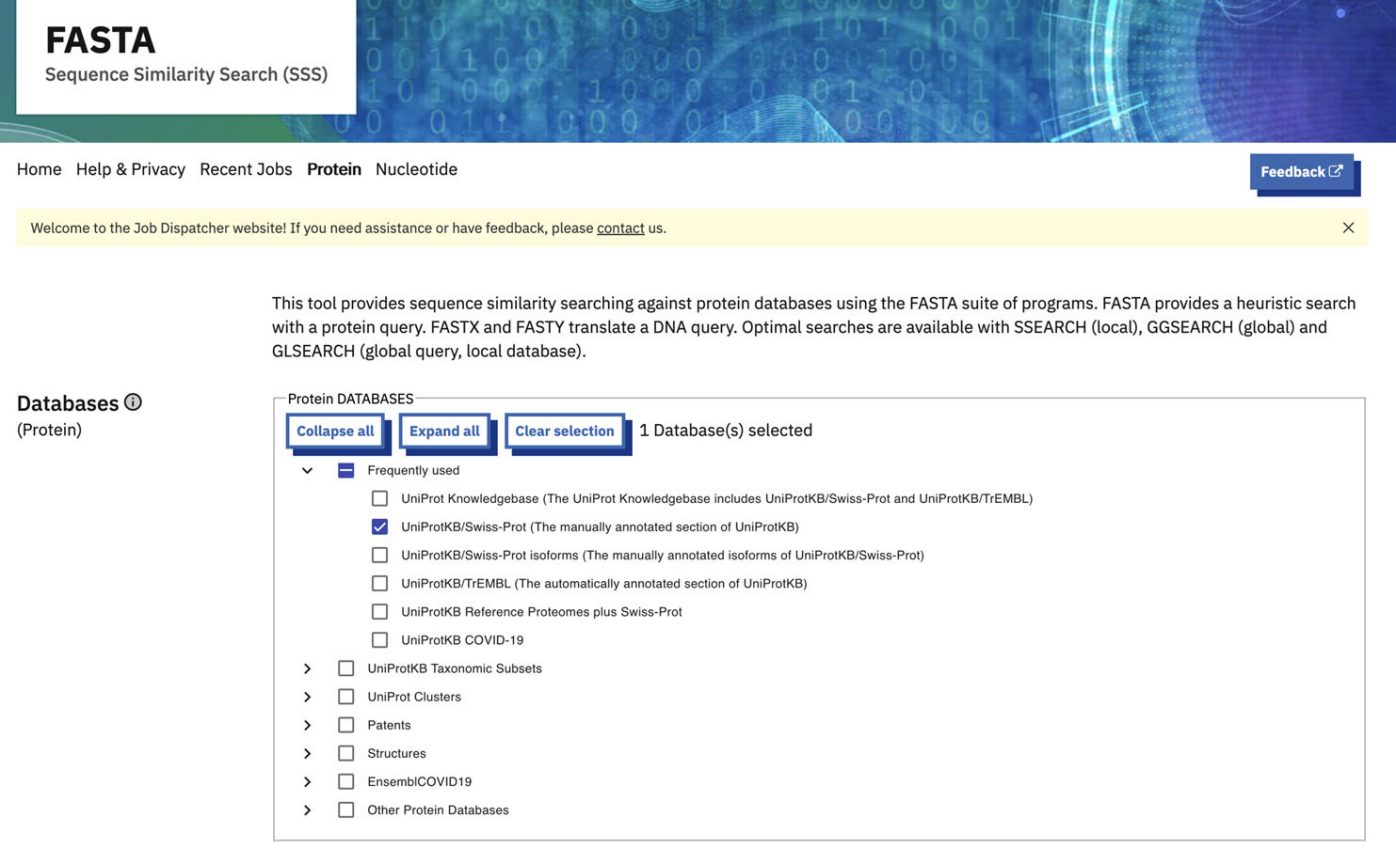
FASTA home page.

2 Under the FASTA section, click ‘Protein’ search or go directly to https://www.ebi.ac.uk/jdispatcher/sss/fasta (Fig. 2).
  In this protocol, we focus on searching against a protein database. Click on ‘Nucleotide’ if the search is to be performed against nucleotide databases.
  The FASTA web form is organized into four sections: i) Databases, where users select the database of interest; multiple databases can be chosen. ii) Input Sequence, where users provide the query sequence. iii) Parameters, where users can set options different from the default values. iv) Submit, where users run the analysis. Users who wish to access documentation can do so by clicking the ‘Help & Privacy’ link located in the top navigation bar.

**Figure 3:**
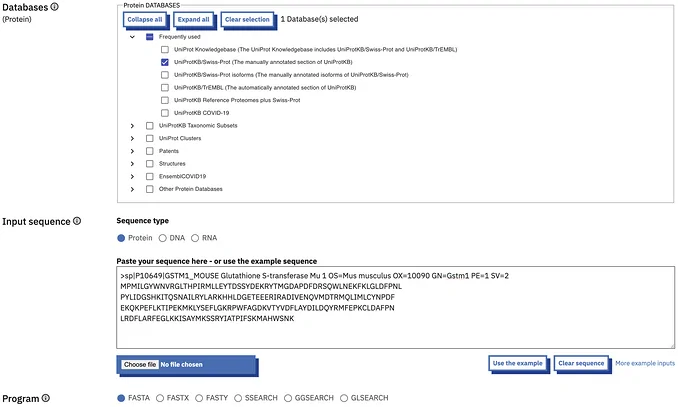
Databases, Input sequence, and Program sections of FASTA search.

3 Select the databases to search.
  In the ‘Databases’ section, choose the database(s) against which you want to search. You can expand or collapse the available databases under each main category by clicking on them, and you can check or uncheck the boxes to select the appropriate databases. It is possible to select multiple databases. For the example depicted here, we select UniProtKB/Swiss‐Prot, which is the default protein database (Fig. 3).

**Figure 4:**
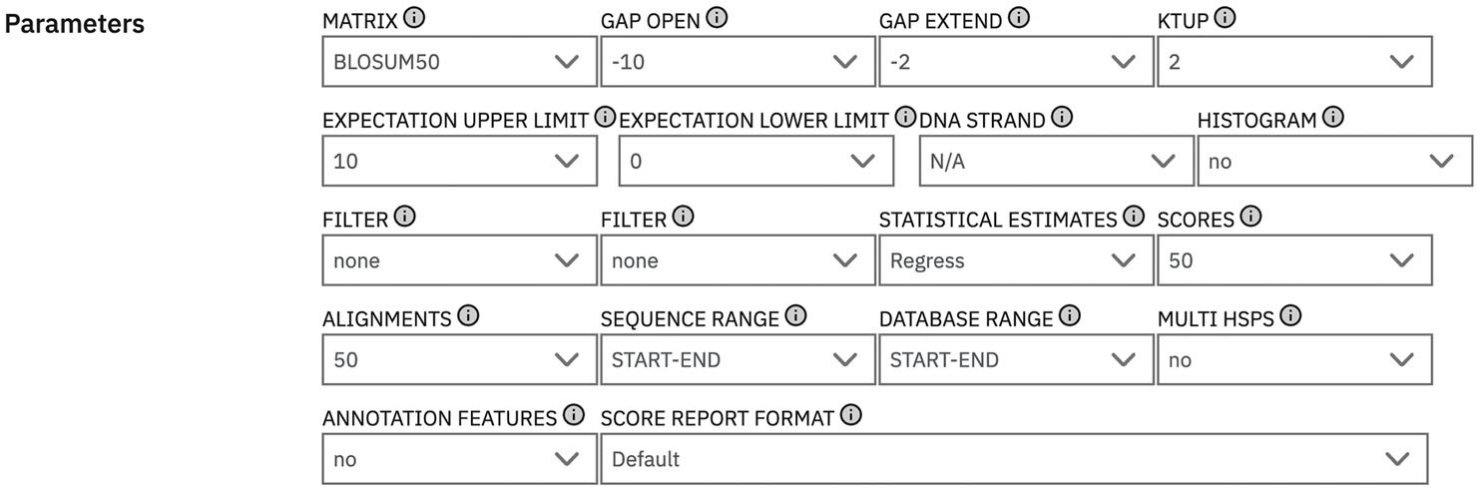
Advanced parameters for FASTA search.

4 Enter the input sequence.
  Upload a sequence file (e.g., Fasta, UniProtKB/Swiss‐Prot) using the ‘Choose file’ button. Alternatively, paste the sequence directly into the sequence text box. You may also use a protein database accession identifier, e.g., UniProtKB:P10649.
  Make sure to select the correct sequence type: Protein for protein sequences or DNA/RNA for nucleotide sequences. In this example, we are using a protein sequence, so Protein is selected (Fig. 3).
5 Choose the FASTA program (Fig. 3).
  To begin the analysis, select the FASTA program that you want to use. The programs available for this search are as follows:
  FASTA: Standard protein similarity search.
  FASTX / FASTY: Translate nucleotide sequences in all reading frames and search against protein databases.
  SSEARCH: Smith‐Waterman alignment; highly sensitive for protein comparisons.
  GLSEARCH / GGSEARCH: Global or semi‐global alignment for full‐length sequence comparison.
  For the example described here, we select FASTA for a standard protein similarity search.
  Before selecting the program, ensure that the correct sequence type is selected (e.g., Protein for protein sequences or DNA/RNA for nucleotide sequences). Changing the program selection may automatically adjust the sequence type, so verify that the correct sequence type is set before proceeding.
6 Set the parameters (Fig. 4).
  Parameters are the settings that control how the search is performed. If you do not change any of these settings, the FASTA tool will use its default parameters, which are suitable for most standard searches.
  Click the ‘More options’ button to expand the advanced parameters section. This includes options for selecting the scoring matrix, gap penalties (gap open and extend), ktup, the expectation value (E‐value) lower limit, multiple high‐scoring segment pairs (HSPs), and more:
  Matrix: Substitution matrix used for scoring (e.g., BLOSUM62, the default for proteins).
  Gap penalties: Gap opening and extension penalties for alignments.
  ktup: Controls the balance between search sensitivity and speed.
  E‐value lower limit: Sets the significance cutoff for reporting matches.
  Multi HSPs: Allows setting more than one local alignment region between the same pair of sequences.
  Adjust the parameter settings to your needs. Tooltips (info icons) provide details about each parameter and are displayed on mouse hover. In this example, we choose the FASTA program and leave the other parameters at their default values.

**Figure 5:**
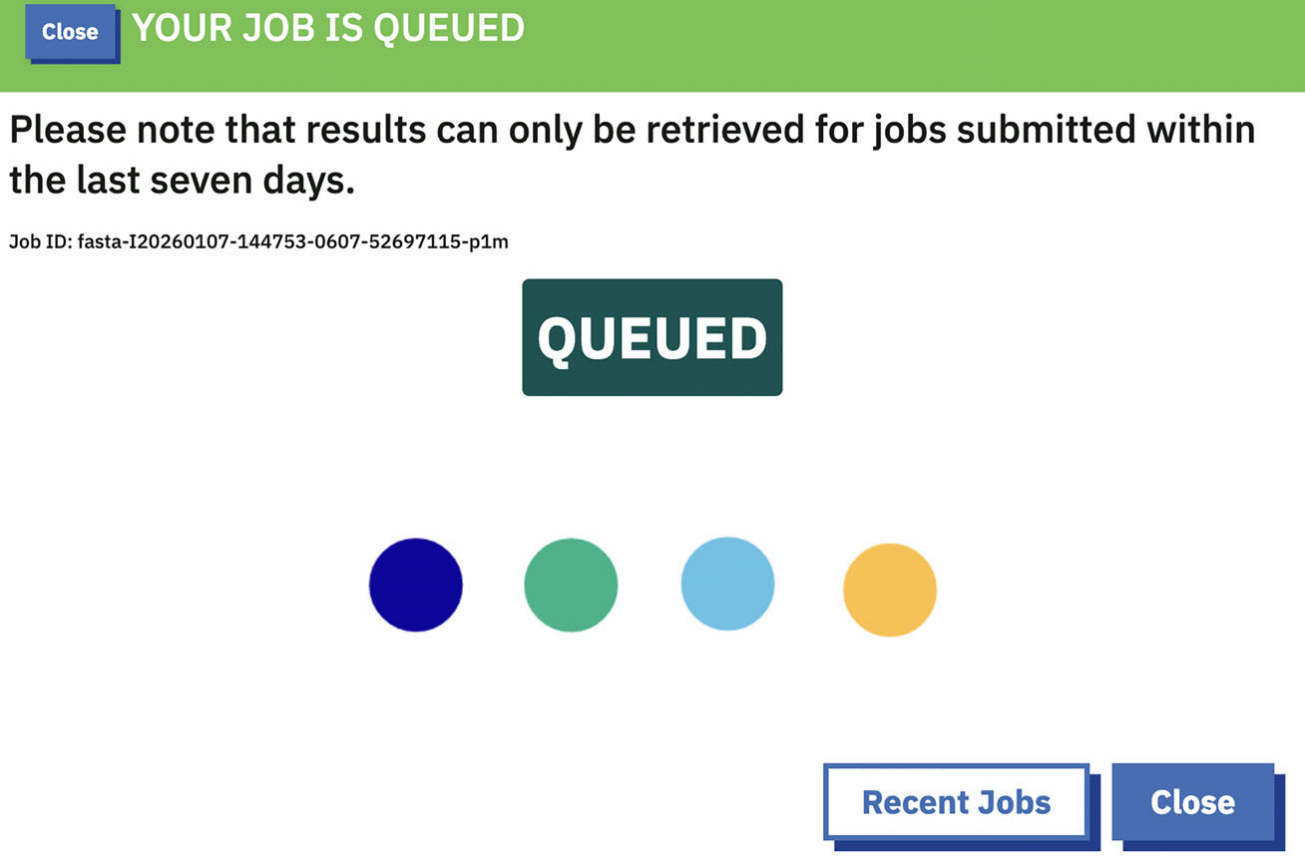
Job ID pop‐up window for FASTA search.

**Figure 6:**
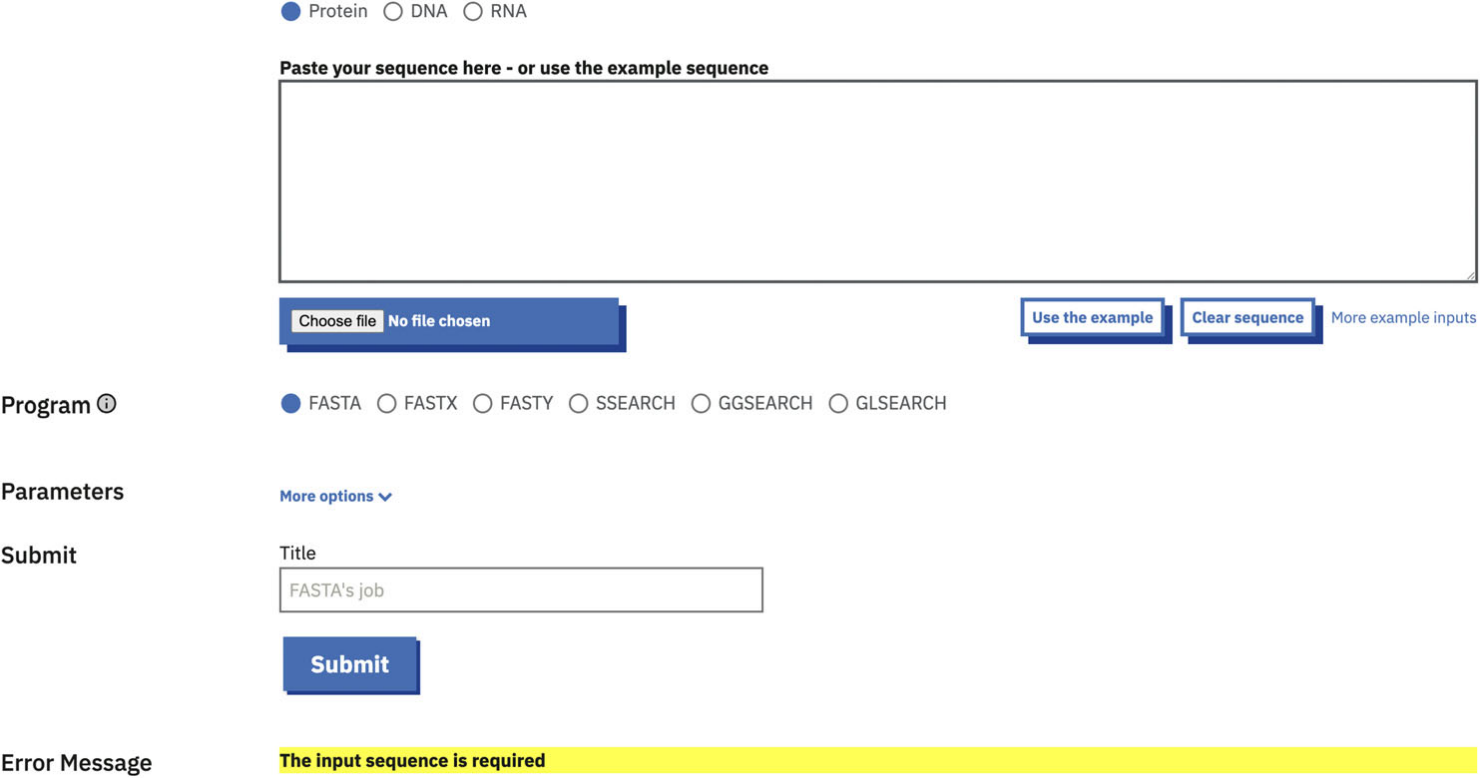
Warnings to provide input sequence for SSS search.

7 Submit the job.
  Once all parameters are set, submit the analysis. Provide an optional Title to briefly describe your job and then click the ‘Submit’ button. A pop‐up window will confirm that your job has been submitted, showing the Job ID and its current status (Fig. 5). The job may be placed in a queue if the servers are busy and will then progress to running and finish, which renders the results page. If any corrections are needed (e.g., missing required fields or invalid parameters), the web form will display warnings (Fig. 6) after you click Submit, prompting you to make the necessary changes.
  You may keep the pop‐up window open to wait for the results or close it to remain on the FASTA homepage to continue with further analysis. You can also navigate to the Recent Jobs page (https://www.ebi.ac.uk/jdispatcher/jobs) to monitor the job progress or return to the results later. This page is particularly useful for long‐running jobs, allowing users to check whether a job has completed or is still in progress. Users can access their submitted jobs on the Recent Jobs page for up to 7 days.

**Figure 7:**
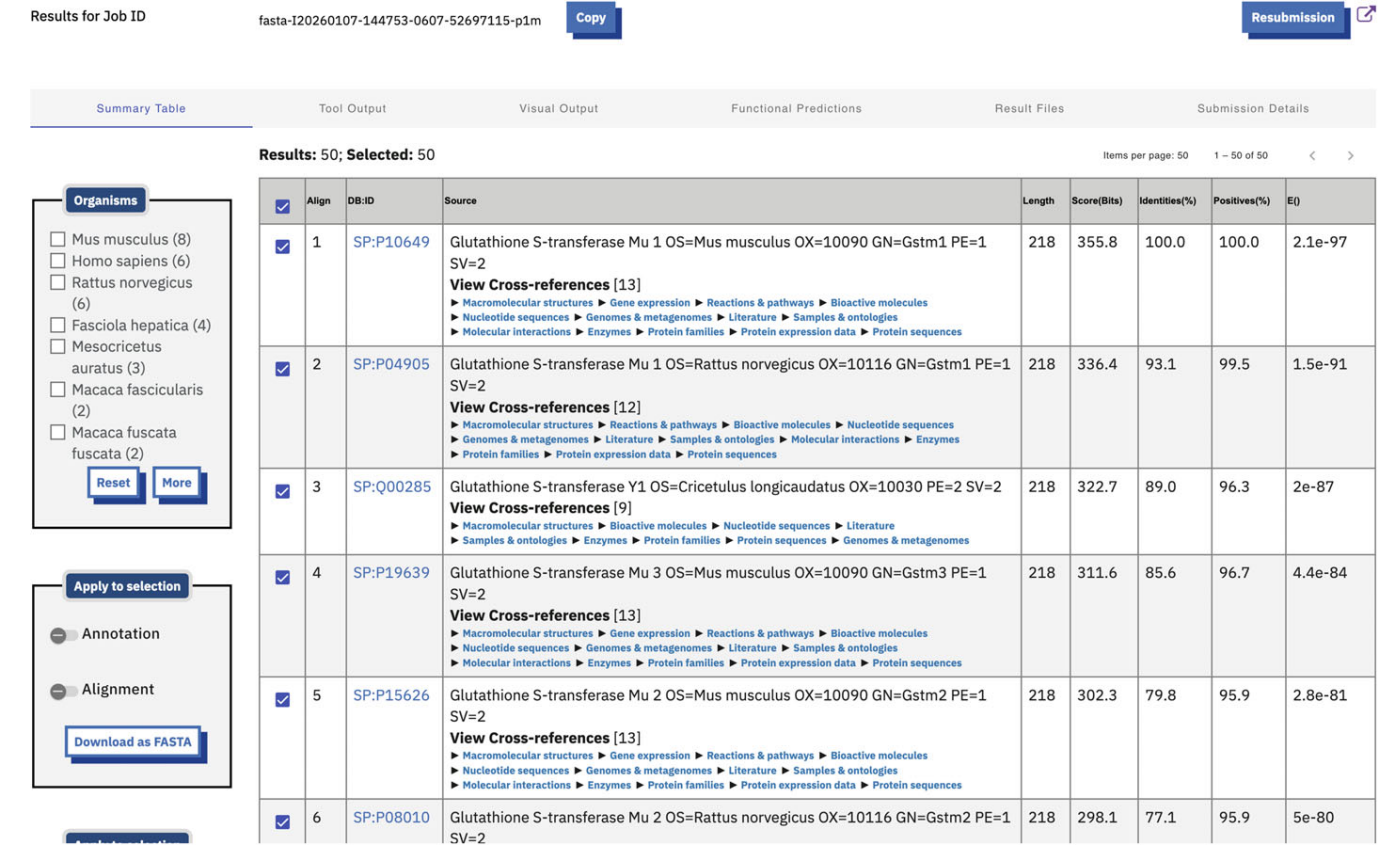
Result Summary Table for FASTA search.

**Figure 8:**
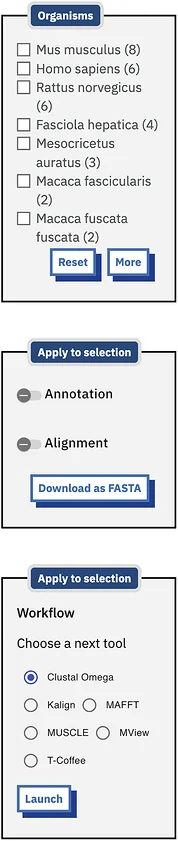
Filter panel for FASTA results page.

8 View job result summary (Fig. 7).
  After submission, the results page will open automatically when the job finishes. Results will be stored on the servers for only 7 days, so users should download any files they wish to keep for future reference.
  The results interface provides several views that may vary across tools but usually remain consistent within a given tool category. For FASTA, the available views include Summary Table, Tool Output, Visual Output, Functional Predictions, Result Files, and Submission Details. The default view displayed is the Summary Table. Use the tabs at the top of the results page to switch between views. The Summary Table lists the top hits returned by the search, including the database, sequence identifier, description, cross‐references, sequence length, bit score, percentage identity, and E‐value. Users can click the sequence identifiers or cross‐reference links to open the corresponding entries in external databases or in EBI Search (Pearce et al., 2025).
  By default, only 50 hits are shown per page; pagination controls allow users to move to the next set of hits.
  A filter panel (Fig. 8) on the left‐hand side allows users to refine the results using available ‘Organisms’ facets, helping to narrow the list of hits to specific organism(s) of interest. Users can also select individual hits by ticking the checkboxes in the first column of the Summary Table. Once you select one or more hits, you can view the annotation or alignments of the selected hits with the query sequence; click ‘Show or hide annotations or alignment.’ An option to download the selected sequences in Fasta format is also available. Additionally, these selected sequences can be directly sent to other MSA tools for further analysis. This supports a seamless workflow from the similarity search to subsequent comparative analyses.

**Figure 9:**
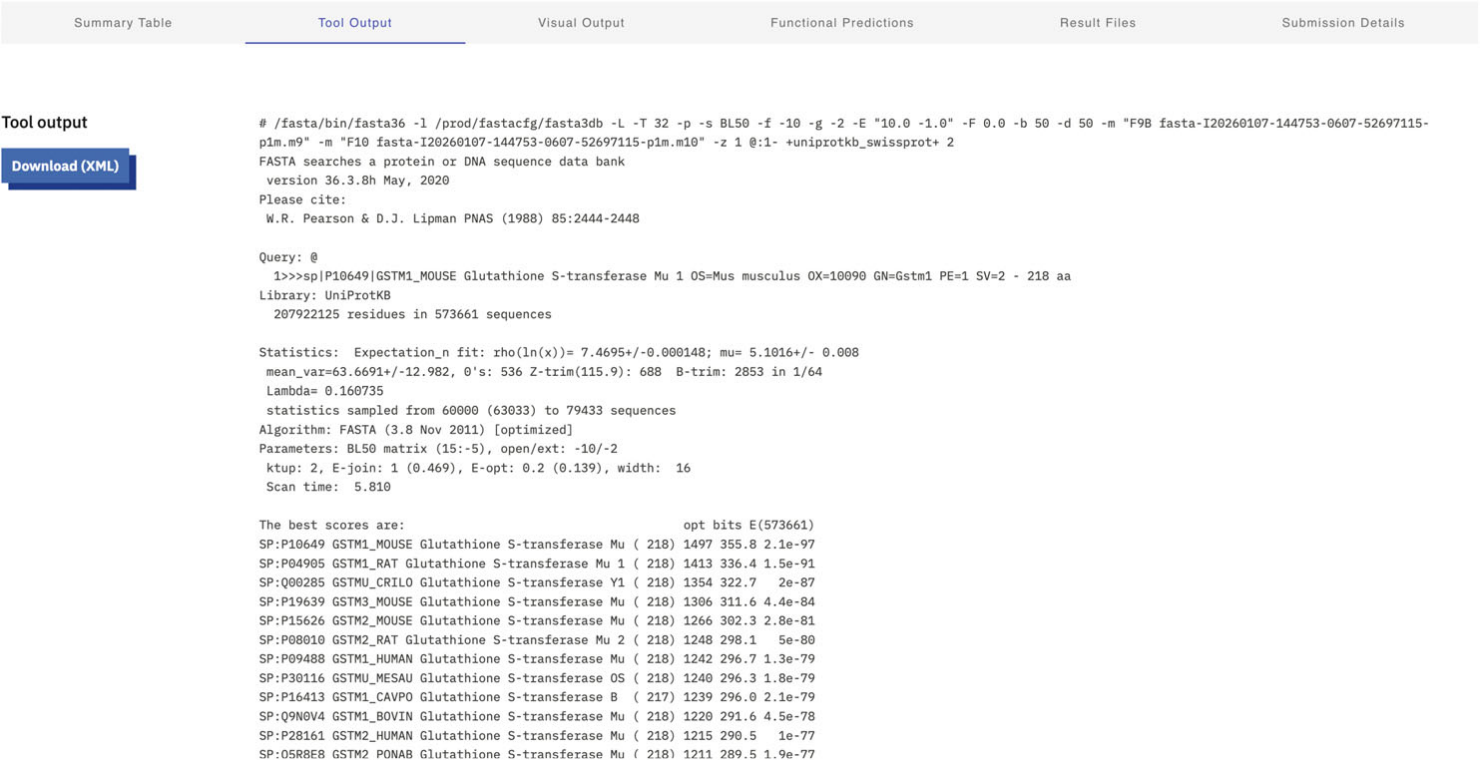
FASTA tool raw output.

9 Display the tool's raw output (Fig. 9).
  Click the Tool Output tab to view the raw output produced by the FASTA program. This includes the complete alignment information for each hit as generated by the tool, showing bit scores, E‐values, and the aligned sequences. The raw output represents the original FASTA results without additional formatting or visualization.

**Figure 10:**
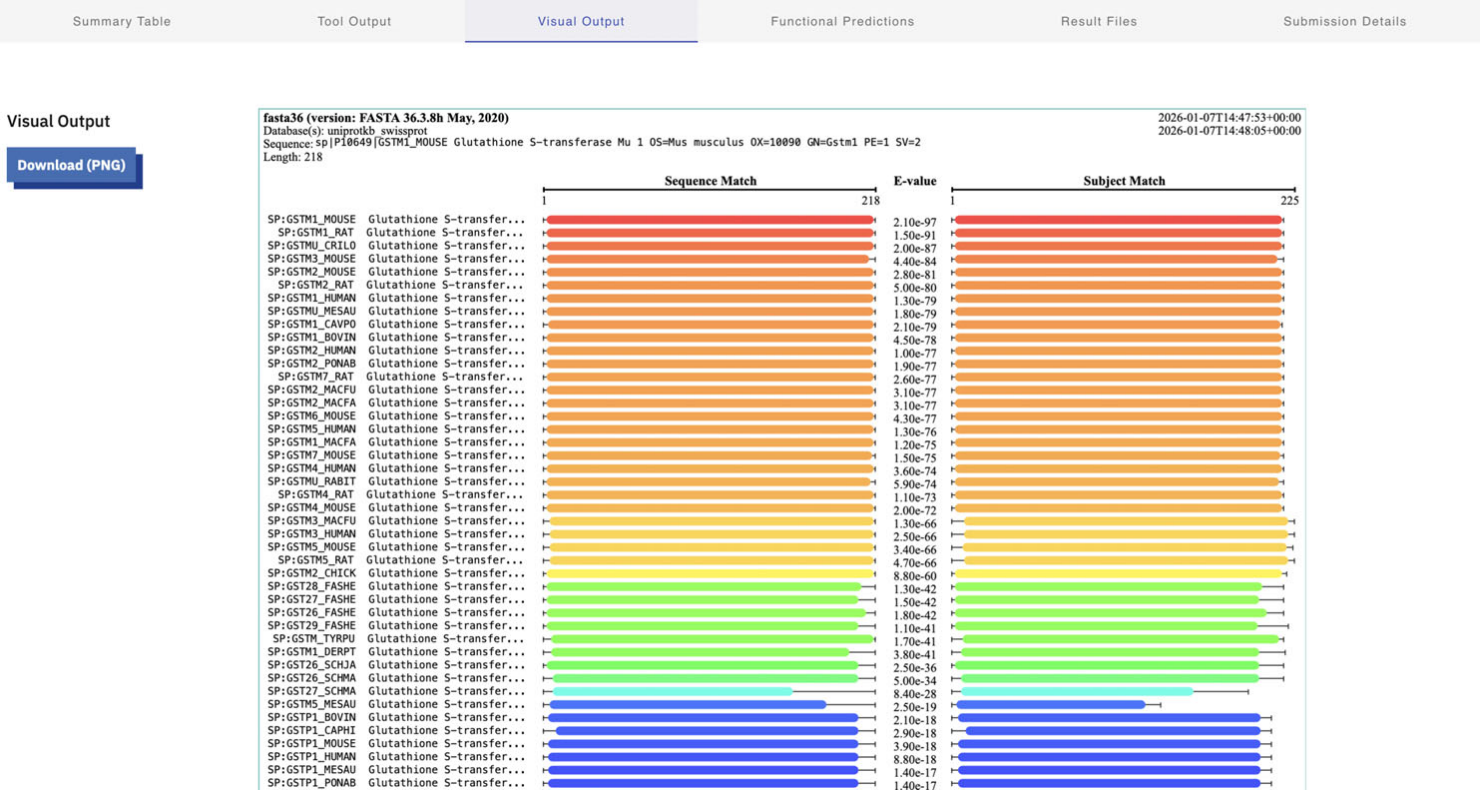
Visual output from FASTA search.

10 Visualize the results (Fig. 10).
  The Visual Output (see Current Protocols article: Madeira, Madhusoodanan, Lee, Eusebi, Niewielska, Tivey, Meacham, et al., 2024; Madeira et al., 2025) shows an interactive graphical view of the results, highlighting top hits, alignment coverage, and conserved regions. Sequence bars are clickable to view additional information and link to external resources, and users can adjust the scale, displayed score (e.g., E‐value, bit score, identity), and coloring scheme to explore the alignments. A static image of the visualization can also be downloaded in PNG format for offline use.

**Figure 11:**
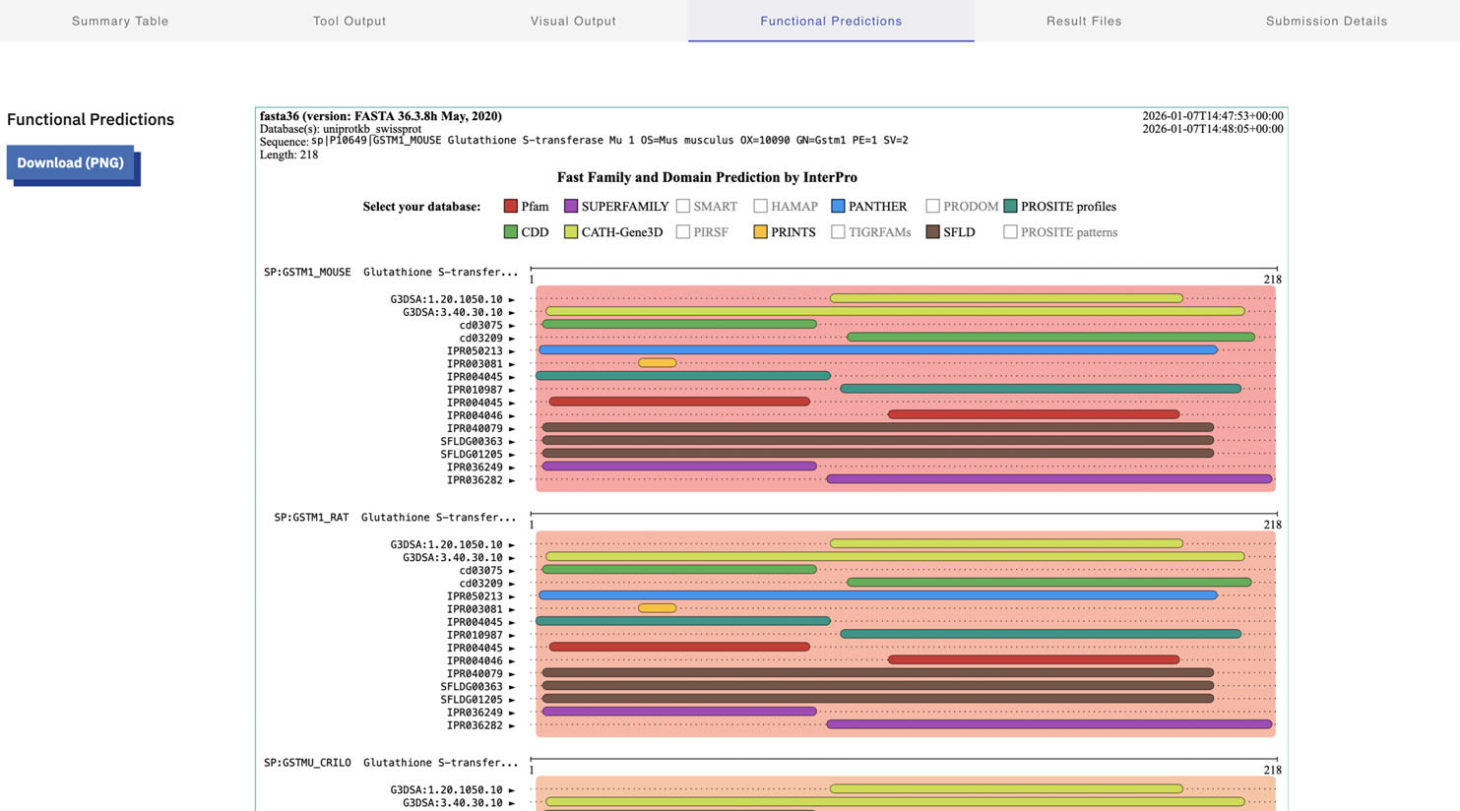
Functional Predictions tab from FASTA search.

11 Display functional predictions (Fig. 11).
  The Functional Predictions view (see Current Protocols article: Madeira, Madhusoodanan, Lee, Eusebi, Niewielska, Tivey, Meacham, et al., 2024; Madeira et al., 2025), available under the Functional Predictions tab on the results page, combines the interactive Visual Output with domain annotations. For each hit, predicted domains from InterPro and its member databases (e.g., Pfam, SUPERFAMILY) are displayed as bars overlaid on the alignment. Users can adjust the scale, score type, and coloring scheme as in the Visual Output and toggle individual domain annotations on and off. A static image of the visualization can also be downloaded in PNG format.

**Figure 12:**
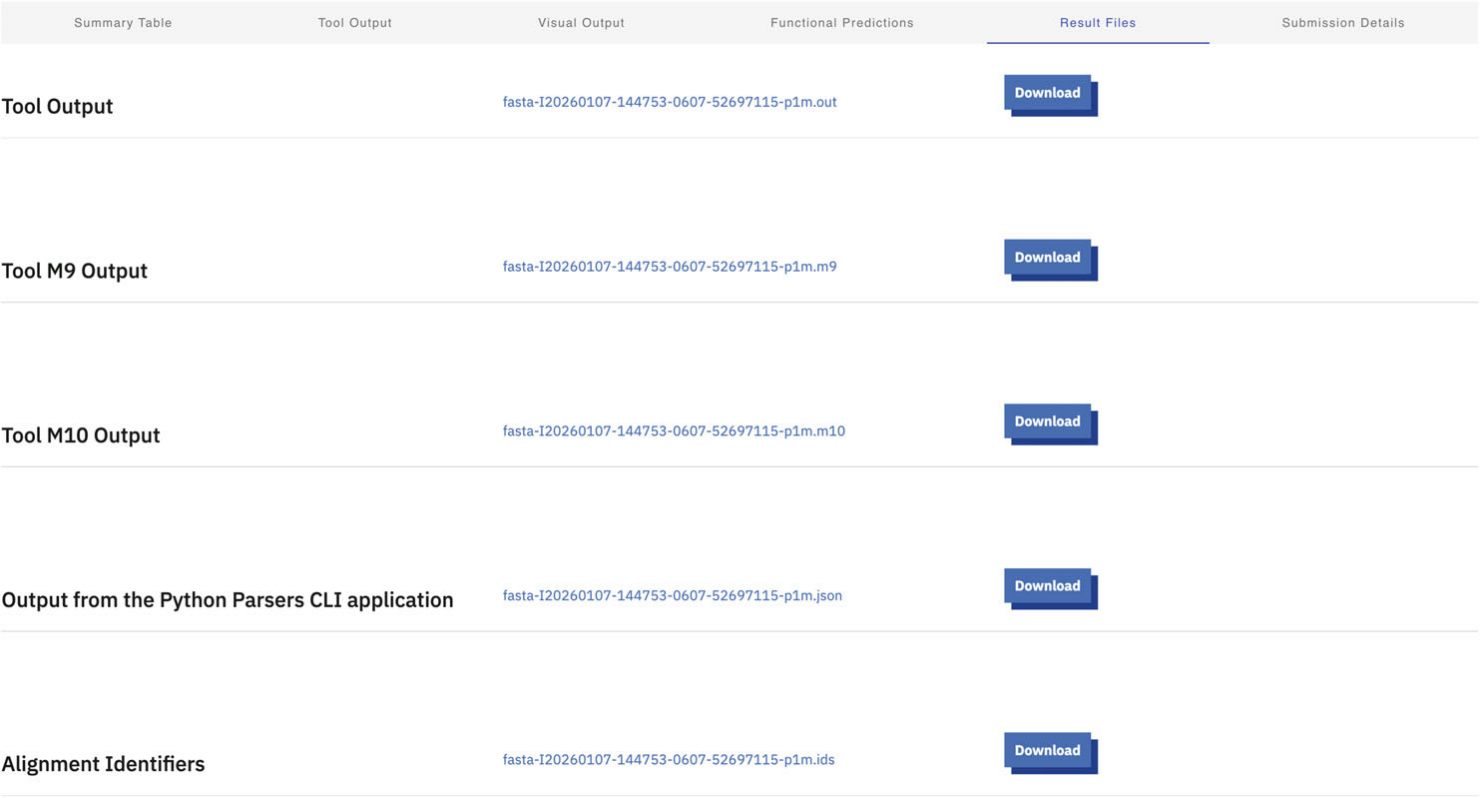
Result Files tab from FASTA search.

12 View Result Files.
  The Result Files tab provides access to all files generated by the tool, including the raw alignment output, a list of alignment accessions and identifiers, XML/JSON‐formatted results, and other visual outputs. Users can download these files individually or as a single ZIP archive for offline analysis or further processing (Fig. 12).

**Figure 13:**
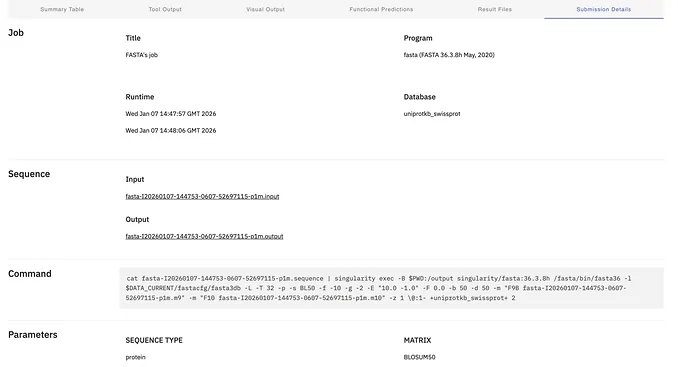
Submission Details tab from FASTA search.

13 Display Submission Details (Fig. 13).
  The Submission Details view provides information about the analysis, including the program and its version, selected database, job title, submission date and time, input and output files, command line executed, and input parameter settings. Users can review these details to verify that the submission is correct and determine whether a resubmission is necessary (see step 14).

14 Resubmit the analysis as needed.
  A new analysis can be prepared by clicking the ‘Resubmission’ button, which starts a new analysis with the current input and settings pre‐filled in the form, allowing you to edit them before submission.

## Basic Protocol 2 SEQUENCE SIMILARITY SEARCH USING NCBI BLAST+

NCBI BLAST+ (Altschul et al., 1997; Camacho et al., 2009; also see Current Protocols article: Ladunga, 2002) emphasizes finding regions of sequence similarity, which will yield functional and evolutionary clues about the structure and function of your novel sequence. Job Dispatcher supports BLAST searches against the protein and nucleotide databases provided by EMBL‐EBI, allowing direct querying of EBI‐curated data. BLAST provides several programs (BLASTP, BLASTN, BLASTX, TBLASTN, and TBLASTX) for protein and nucleotide searches, enabling flexible analysis depending on the query type and database. In this protocol, we focus on performing a protein BLAST (BLASTP) search against a protein database using the EMBL‐EBI Job Dispatcher web interface.

### Necessary Resources

#### Hardware

Any computer with internet access.

##### Software

A modern web browser (e.g., Chrome, Firefox, Edge, Safari).

##### Input

A nucleotide or protein sequence in a supported format (e.g., Fasta, EMBL, GenBank, PHYLIP, UniProtKB/Swiss‐Prot) can be pasted into the form or uploaded.
Alternatively, a database identifier in the format database:identifier can be used (e.g., UniProtKB:GSTM1_MOUSE).

##### Example for this protocol

The mouse protein Glutathione S‐transferase Mu 1 (UniProtKB:P10649) will be used as input:
  Entry details: https://www.uniprot.org/uniprotkb/P10649/entry
  Fasta‐format sequence: https://rest.uniprot.org/uniprotkb/P10649.fasta

**Figure 14:**
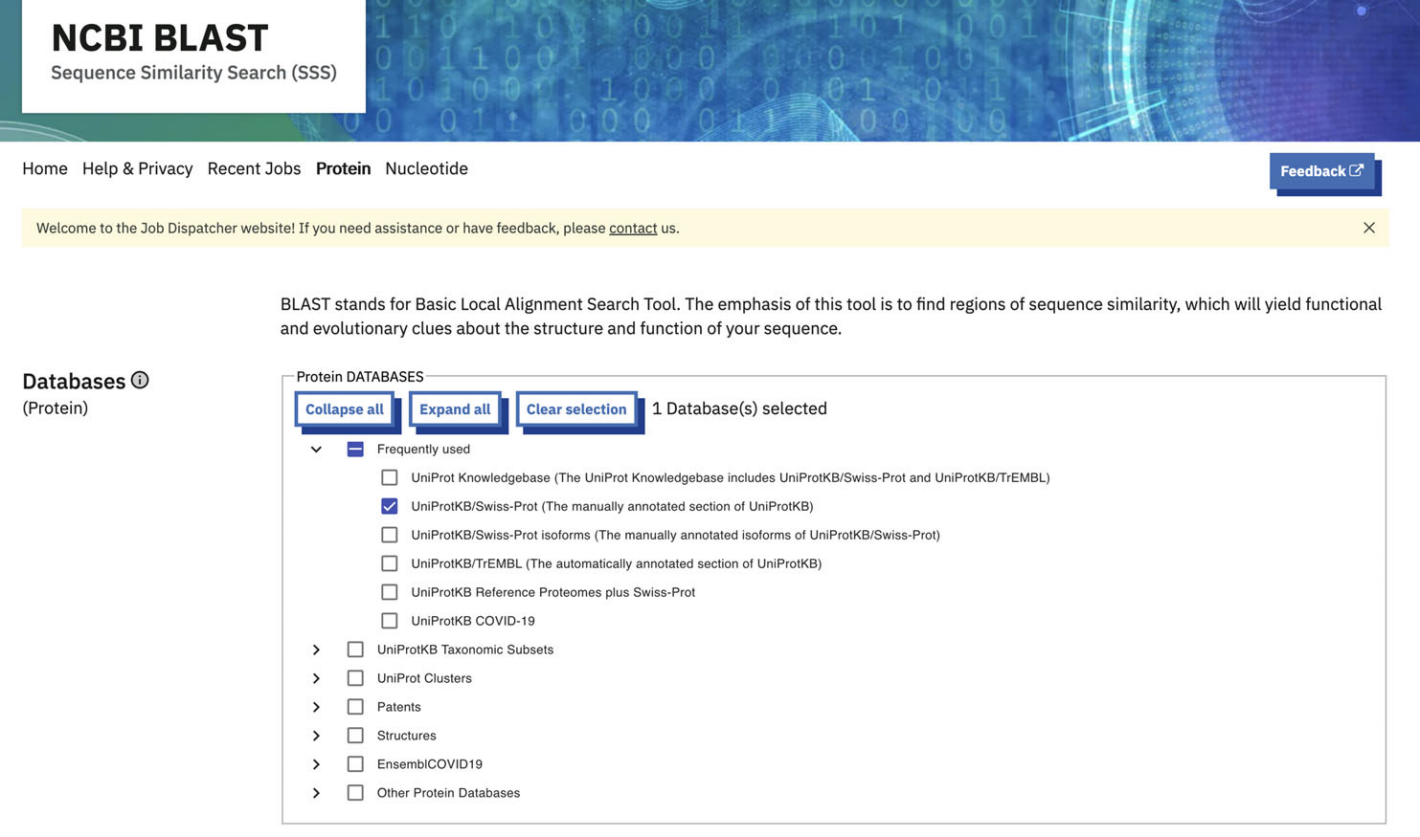
BLAST home page.

1 Open a web browser and go to the SSS page https://www.ebi.ac.uk/jdispatcher/sss using a web browser (Fig. 1).
  This page lists available tools for protein and nucleotide SSSs.
2 Under the NCBI BLAST section, click ‘Protein’ search or go directly to https://www.ebi.ac.uk/jdispatcher/sss/ncbiblast (Fig. 14).
  In this protocol, we focus on searching against a protein database. Click on ‘Nucleotide’ if the search is to be performed against nucleotide databases.
  The BLAST web form is organized into four sections: i) Databases, where users select the database or databases of interest; multiple databases can be chosen. ii) Input Sequence, where users provide the query sequence. Iii) Parameters, where users can set options different from the default values. iv) Submit, where users run the analysis. Users who wish to access documentation can do so by clicking the ‘Help & Privacy’ link located in the top navigation bar.

**Figure 15:**
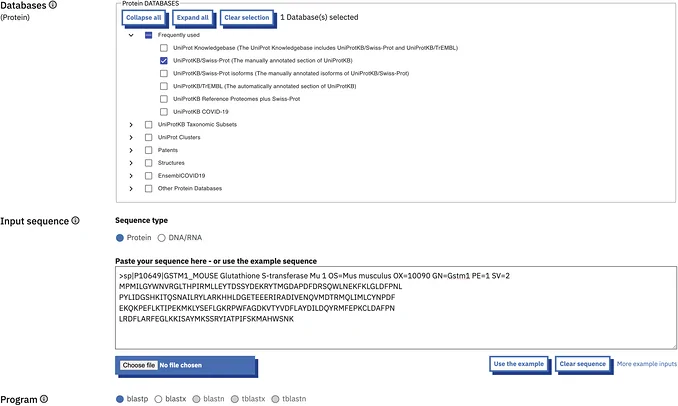
Databases, Input sequence, and Program sections of BLAST search.

3 Select the databases to search.
  In the ‘Databases’ section, choose the database(s) against which you want to search. You can expand or collapse the available databases under each main category by clicking on them, and you can check or uncheck the boxes to select the appropriate databases. It is possible to select multiple databases. For the example depicted here, we select UniProtKB/Swiss‐Prot, which is the default protein database (Fig. 15).

**Figure 16:**
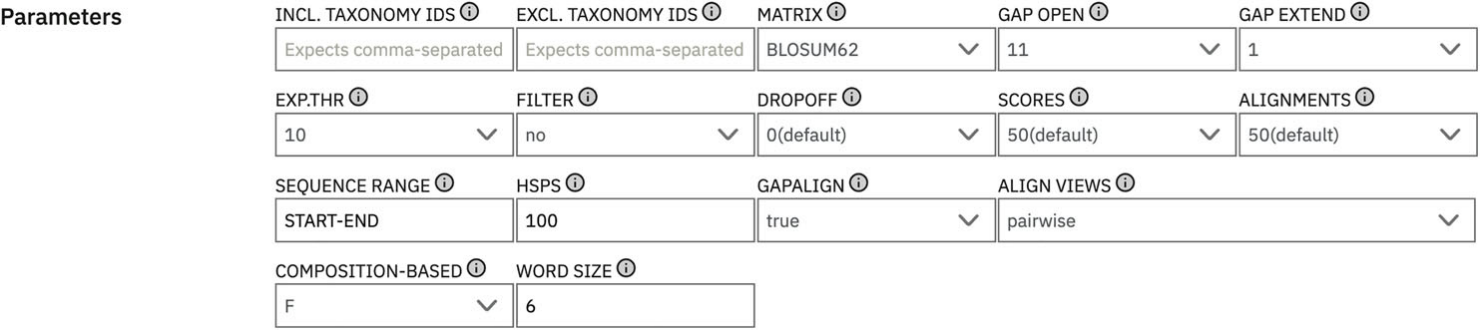
Advanced parameters for BLAST search.

4 Enter the input sequence.
  Upload a sequence file (e.g., Fasta, UniProtKB/Swiss‐Prot) using the ‘Choose file’ button. Alternatively, paste the sequence directly into the sequence text box. You may also use a protein database accession identifier, e.g., UniProtKB:P10649.
  Make sure to select the correct sequence type: Protein for protein sequences or DNA/RNA for nucleotide sequences. In this example, we are using a protein sequence, so Protein is selected (Fig. 15).
5 Choose the BLAST program (Fig. 15).
  To begin the analysis, select the BLAST program that you want to use. The programs available for this search are as follows:
  BLASTP: Protein vs. protein database.
  BLASTN: Nucleotide vs. nucleotide database.
  BLASTX: Translate nucleotide query to protein and search against protein database.
  TBLASTN: Protein query vs. translated nucleotide database.
  TBLASTX: Translated nucleotide query vs. translated nucleotide database.
  For the example described here, we select BLASTP for a standard protein similarity search.
  Before selecting the program, ensure that the correct sequence type is selected (e.g., Protein for protein sequences or DNA/RNA for nucleotide sequences). Changing the program selection may automatically adjust the sequence type, so verify that the correct sequence type is set before proceeding.
6 Set the parameters (Fig. 16).
  Parameters are the settings that control how the search is performed. If you do not change any of these settings, the BLAST tool will use its default parameters, which are suitable for most standard searches.
  Click on the ‘More options’ button to expand the section for the advanced parameters, which include include/exclude taxonomy, matrix, gap penalties, and E‐value threshold, among others:
  Include/exclude taxonomy: Restrict or focus the search to specific organisms or taxonomic groups. Type the NCBI Taxon ID(s) to include or exclude. Multiple IDs can be entered, separated by commas (no spaces). Currently, only the UniProtKB and ENA databases support filtering by Taxonomy ID.
  Matrix: Substitution matrix used for scoring (e.g., BLOSUM62, the default for proteins).
  Gap penalties: Gap opening and extension penalties for alignments.
  Exp.Thr (E‐value threshold): Maximum expected hits to report.
  Adjust the parameter settings to your needs. Tooltips (info icons) provide details about each parameter and are displayed on mouse hover. In this example, we choose the BLASTP program and leave the other parameters at their default.

**Figure 17:**
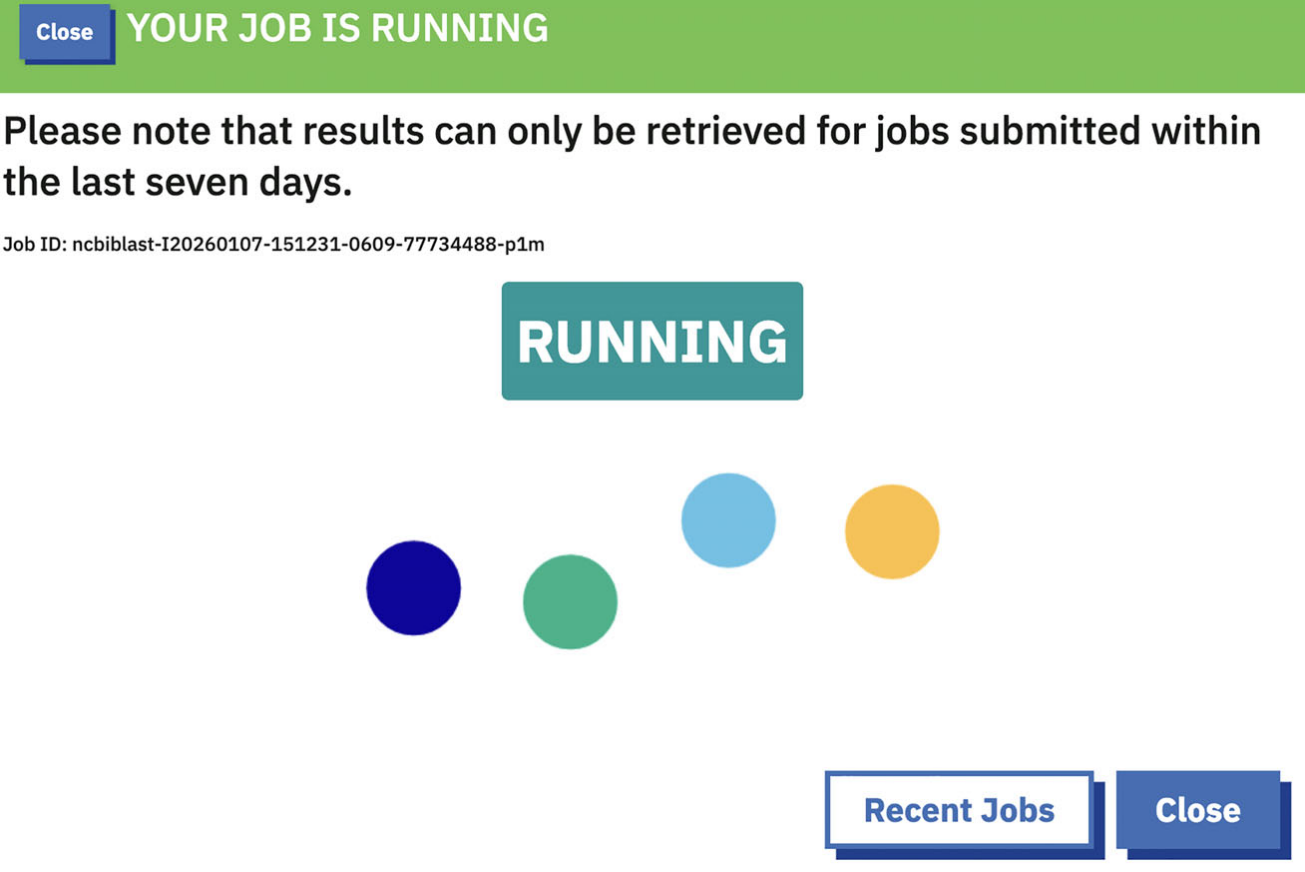
Job ID pop‐up window for BLAST search.

7 Submit the job.
  Once all parameters are set, submit the analysis. Provide an optional Title to briefly describe your job, and then click the ‘Submit’ button. A pop‐up window will confirm that your job has been submitted, showing the Job ID and its current status (Fig. 17). The job may be placed in a queue if the servers are busy and will then progress to running and finish, which renders the results page. If any corrections are needed (e.g., missing required fields or invalid parameters), the web form will display warnings (Fig. 6) after you click Submit, prompting you to make the necessary changes.
  You may keep the pop‐up window open to wait for the results or close it to remain on the BLAST homepage to continue with further analysis. You can also navigate to the Recent Jobs page (https://www.ebi.ac.uk/jdispatcher/jobs) to monitor the job progress or return to the results later. This page is particularly useful for long‐running jobs, allowing users to check whether a job has completed or is still in progress. Users can access each of their job submissions on the Recent Jobs page for up to 7 days from the time the jobs were submitted.

**Figure 18:**
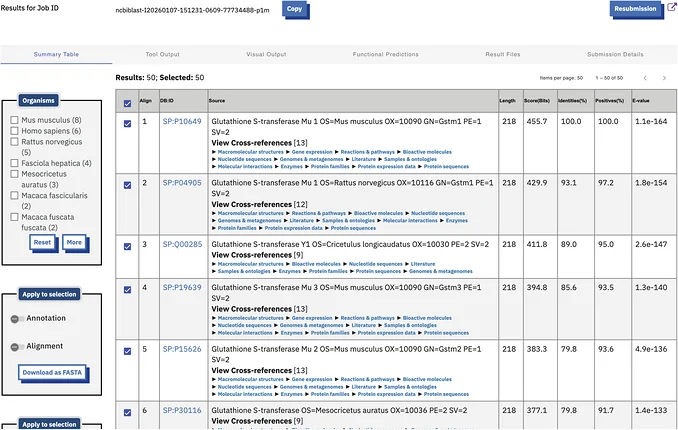
Result Summary Table for BLAST search.

**Figure 19:**
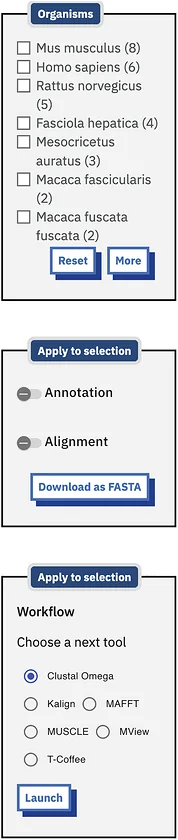
Filter panel for BLAST results page.

8 View job result summary (Fig. 18).
  After submission, the results page will open automatically when the job finishes. Results will be stored on the servers for only 7 days, so users should download any files they wish to keep for future reference.
  The results interface provides several views that may vary across tools but usually remain consistent within a given tool category. For BLAST, the available views include Summary Table, Tool Output, Visual Output, Functional Predictions, Result Files, and Submission Details. The default view displayed is the Summary Table. Use the tabs at the top of the results page to switch between views. The Summary Table lists the top hits returned by the search, including the database, sequence identifier, sequence length, bit score, percentage identity, E‐value, description, and cross‐references. Users can click the sequence identifiers or cross‐reference links to open the corresponding entries in external databases or in EBI Search (Pearce et al., 2025).
  By default, only 50 hits are shown per page; pagination controls allow users to move to the next set of hits.
  A filter panel (Fig. 19) on the left‐hand side allows users to refine the results using available ‘Organisms’ facets, helping to narrow the list of hits to specific organism(s) of interest. Users can also select individual hits by ticking the checkboxes in the first column of the Summary Table. Once you select one or more hits, you can view the annotation or alignments of the selected hits with the query sequence; click ‘Show or hide annotations or alignment.’ An option to download the selected sequences in Fasta format is also available. Additionally, these selected sequences can be directly sent to other MSA tools for further analysis. This supports a seamless workflow from the similarity search to subsequent comparative analyses.

**Figure 20:**
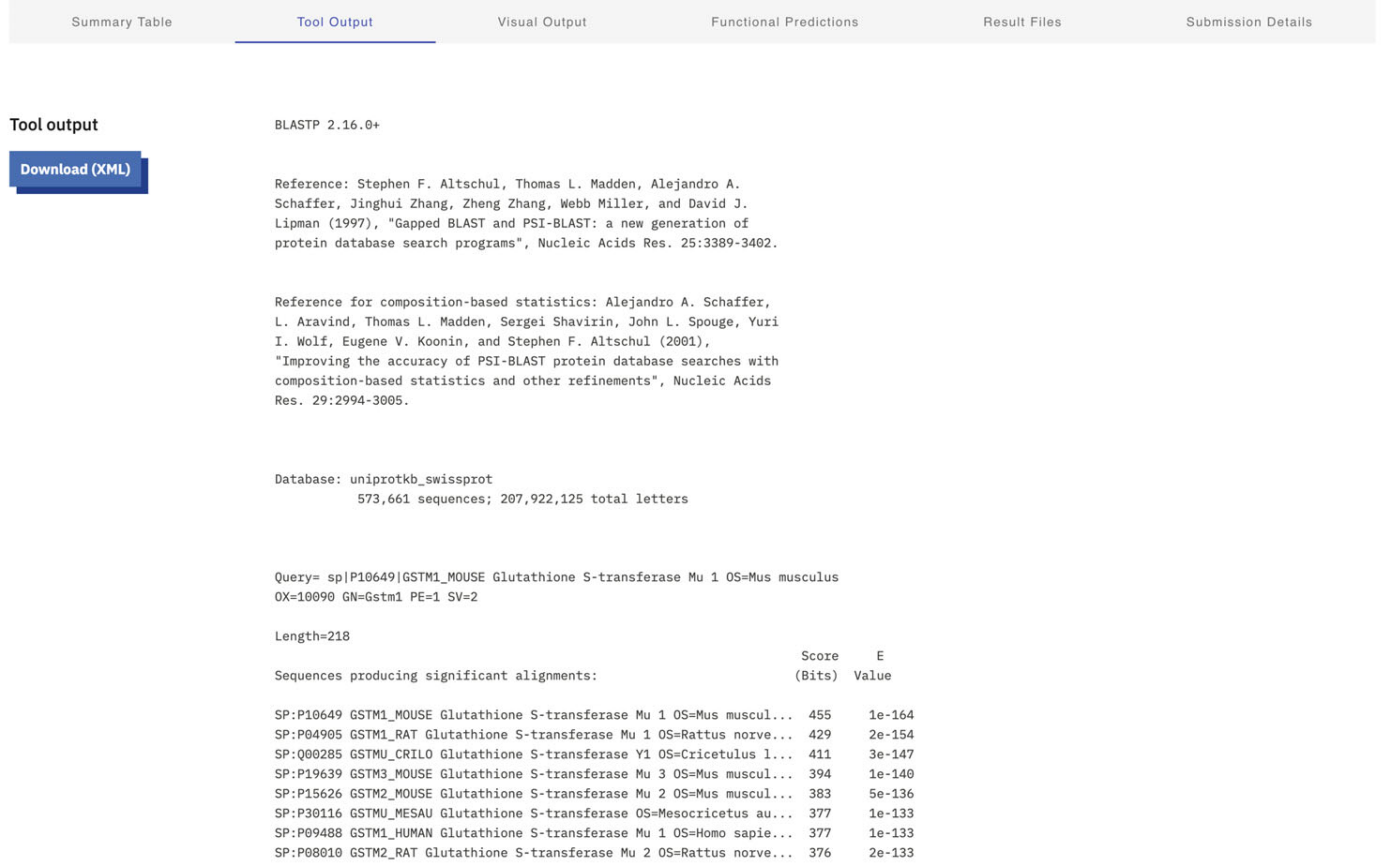
BLAST tool raw output.

9 Display the tool's raw output (Fig. 20).
  Click the Tool Output tab to view the raw output produced by the BLAST program. This includes the complete alignment information for each hit as generated by the tool, showing bit scores, E‐values, and the aligned sequences. The raw output represents the original BLAST results without additional formatting or visualization.

**Figure 21:**
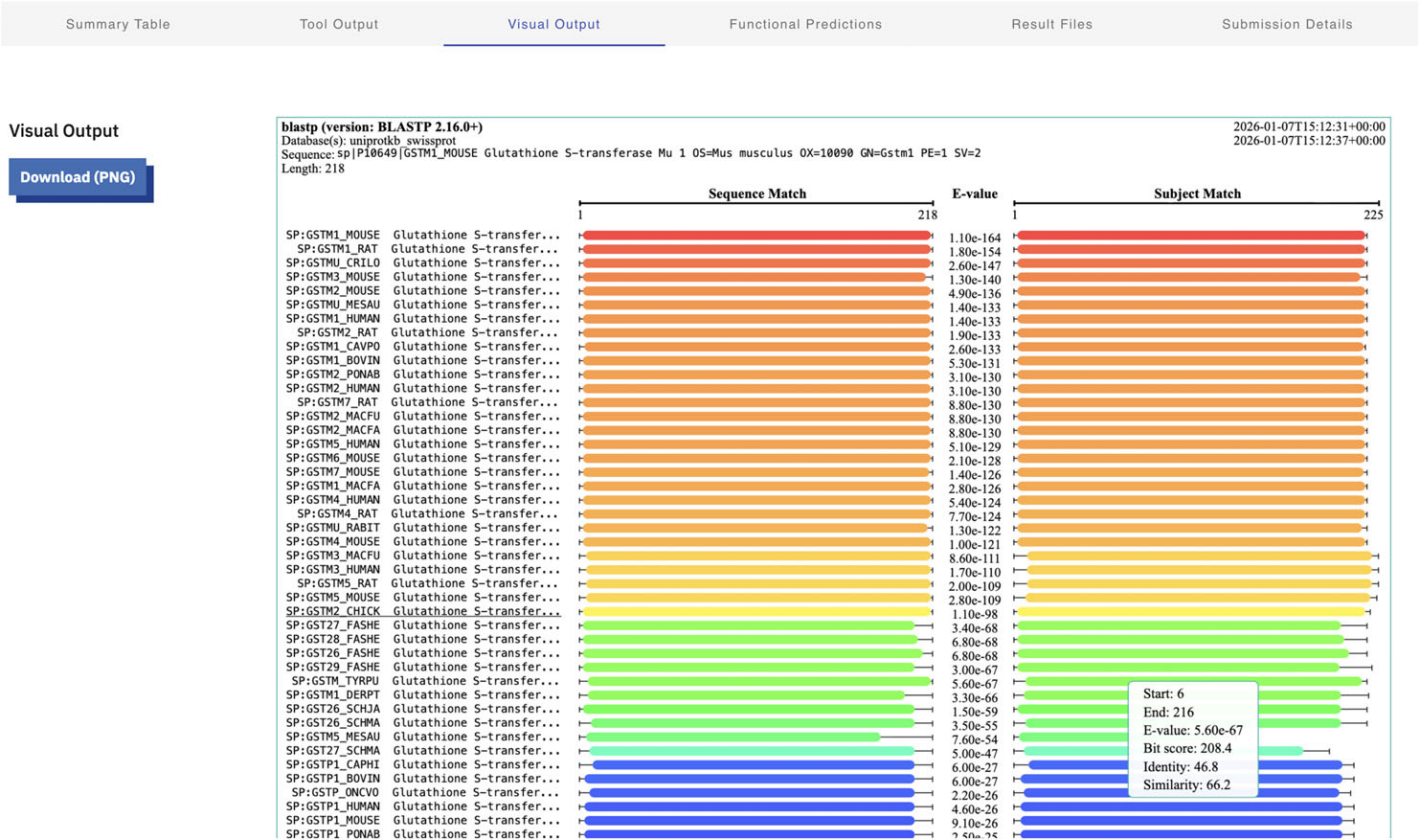
Visual output from BLAST search.

10 Visualize the results (Fig. 21).
  The Visual Output (see Current Protocols article: Madeira, Madhusoodanan, Lee, Eusebi, Niewielska, Tivey, Meacham, et al., 2024; Madeira et al., 2025) shows an interactive graphical view of the results, highlighting top hits, alignment coverage, and conserved regions. Sequence bars are clickable to view additional information and link to external resources, and users can adjust the scale, displayed score (e.g., E‐value, bit score, identity), and coloring scheme to explore the alignments. A static image of the visualization can also be downloaded in PNG format for offline use.

**Figure 22:**
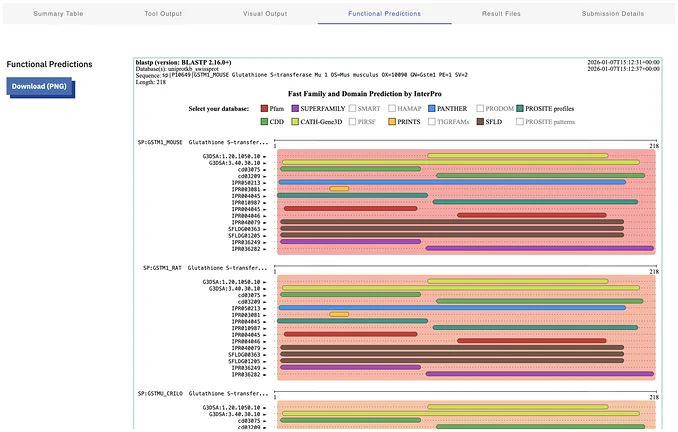
Functional Predictions tab from BLAST search.

11 Display functional predictions (Fig. 22).
  The Functional Predictions view (see Current Protocols article: Madeira, Madhusoodanan, Lee, Eusebi, Niewielska, Tivey, Meacham, et al., 2024; Madeira et al., 2025), available under the Functional Predictions tab on the results page, combines the interactive Visual Output with domain annotations. For each hit, predicted domains from InterPro and its member databases (e.g., Pfam, SUPERFAMILY) are displayed as bars overlaid on the alignment. Users can adjust the scale, score type, and coloring scheme as in the Visual Output and toggle individual domain annotations on and off. A static image of the visualization can also be downloaded in PNG format.

**Figure 23:**
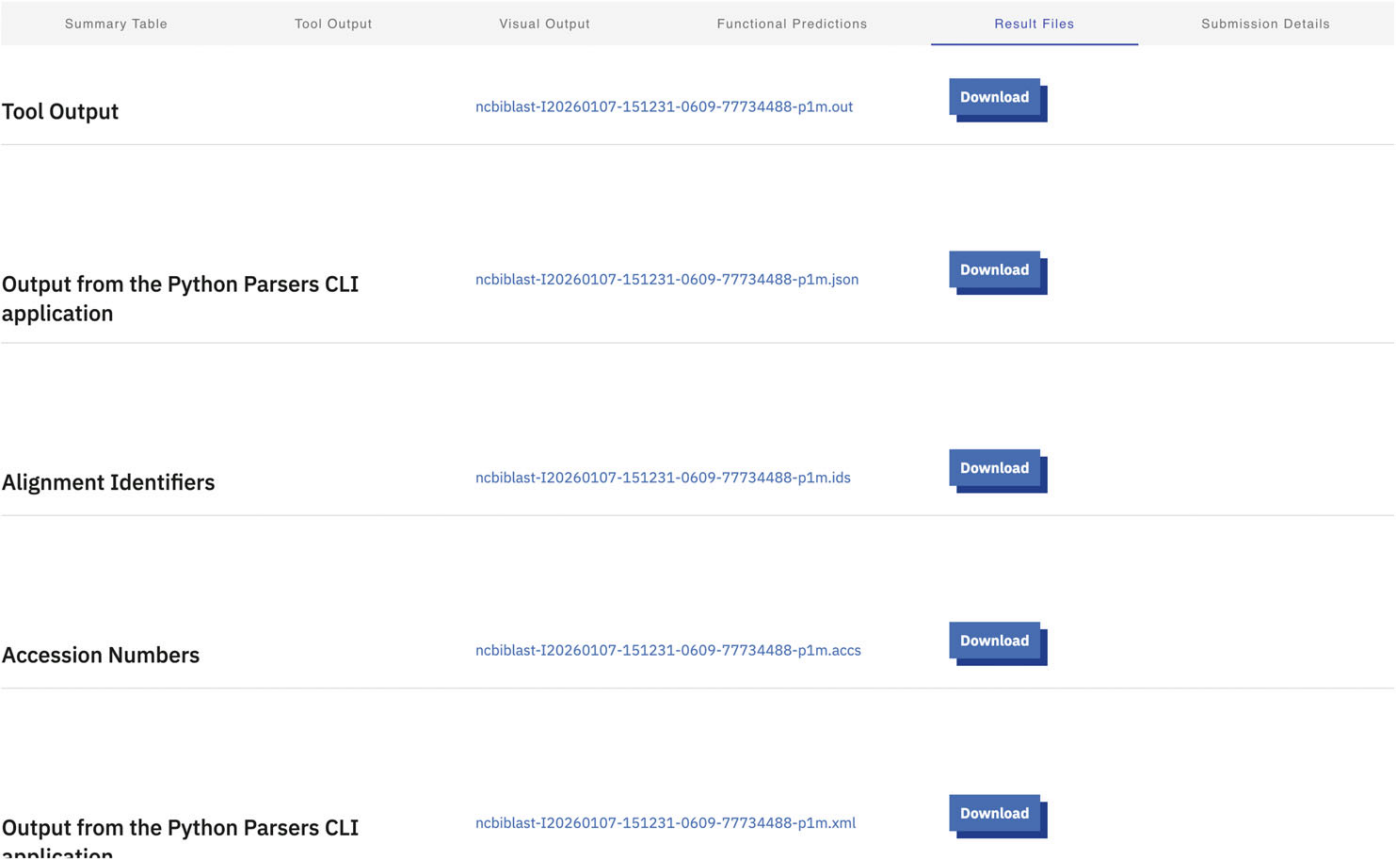
Result Files tab from BLAST search.

12 View Result Files.
  The Result Files tab provides access to all files generated by the tool, including the raw alignment output, a list of alignment accessions and identifiers, XML/JSON‐formatted results, and other visual outputs. Users can download these files individually or as a single ZIP archive for offline analysis or further processing (Fig. 23).

**Figure 24:**
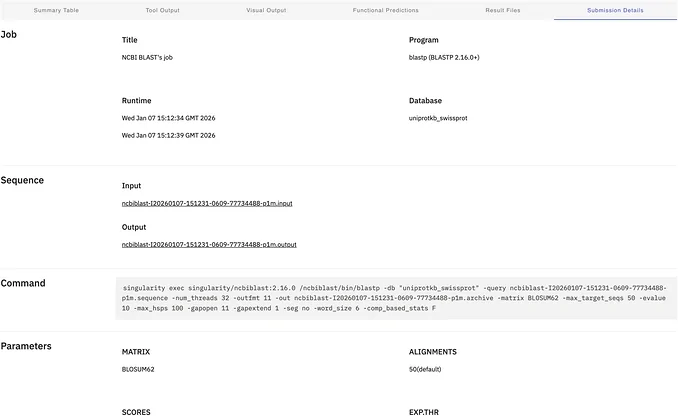
Submission Details tab from BLAST search.

13 Display Submission Details (Fig. 24).
  The Submission Details view provides information about the analysis, including the program and its version, selected database, job title, submission date and time, input and output files, command line executed, and input parameter settings. Users can review these details to verify that the submission is correct and determine whether a resubmission is necessary (see step 14).

14 Resubmit the analysis as needed.
  A new analysis can be prepared by clicking the ‘Resubmission’ button, which starts a new analysis with the current input and settings pre‐filled in the form, allowing you to edit them before submission.

## Basic Protocol 3 SEQUENCE SIMILARITY SEARCH USING PSI‐BLAST

PSI‐BLAST (Position‐Specific Iterated BLAST) (Altschul et al., 1997; Camacho et al., 2009; also see Current Protocols article: Ladunga, 2002) identifies distant homologs by iteratively building a position‐specific scoring matrix (PSSM). Unlike standard BLASTP, PSI‐BLAST increases sensitivity with each iteration by incorporating significant hits into a profile. This makes it particularly powerful for detecting remote evolutionary relationships, weak similarities, and divergent protein families.

The EMBL‐EBI Job Dispatcher provides a web interface to run PSI‐BLAST searches without requiring command‐line expertise and search against EMBL‐EBI databases. This protocol explains how to launch a PSI‐BLAST job, refine parameters, and interpret and iterate results.

### Necessary Resources

#### Hardware

Any computer with internet access.

##### Software

A modern web browser (e.g., Chrome, Firefox, Edge, Safari).

##### Input

A protein sequence in a supported format (e.g., Fasta, EMBL, GenBank, PHYLIP, UniProtKB/Swiss‐Prot) can be pasted into the form or uploaded.
Alternatively, a database identifier in the format database:identifier can be used (e.g., UniProtKB:GSTM1_MOUSE).

##### Example for this protocol

The mouse protein Glutathione S‐transferase Mu 1 (UniProtKB:P10649) will be used as input:
  Entry details: https://www.uniprot.org/uniprotkb/P10649/entry
  Fasta‐format sequence: https://rest.uniprot.org/uniprotkb/P10649.fasta

**Figure 25:**
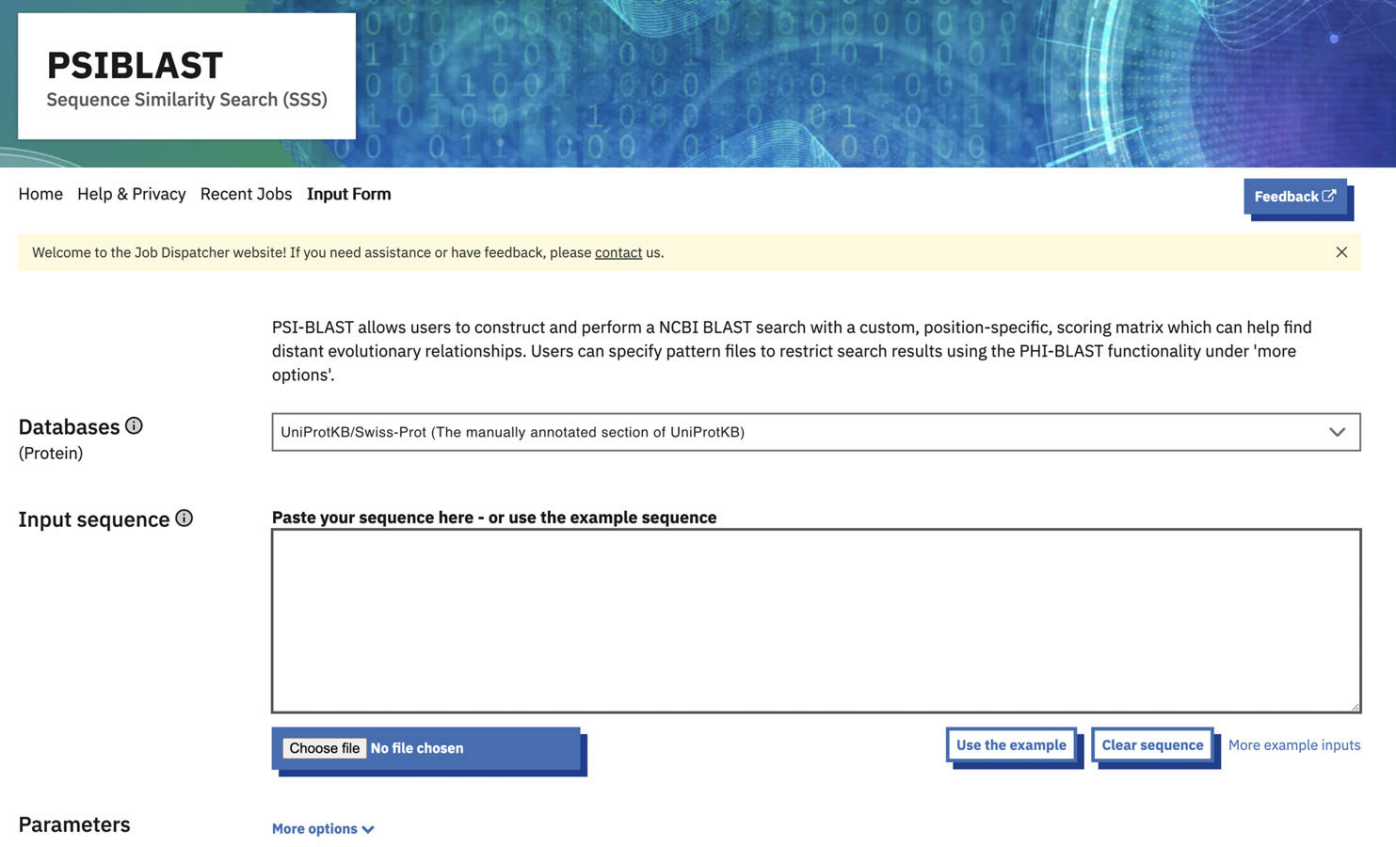
PSI‐BLAST home page.

1 Open a web browser and go to the SSS page https://www.ebi.ac.uk/jdispatcher/sss using a web browser (Fig. 1).
  This page lists available tools for protein and nucleotide SSSs.
2 Under the PSI‐BLAST section, click ‘Protein’ search or go directly to https://www.ebi.ac.uk/jdispatcher/sss/psiblast (Fig. 25).
  PSI‐BLAST is an iterative variant of BLAST designed to detect distant homologs of protein sequences. It only accepts protein sequences as input and searches a single protein database.
  The PSI‐BLAST web form is organized into four sections: i) Databases, where users select the database to search against. ii) Input Sequence, where users provide the query sequence. iii) Parameters, where users can set options different from the default values. iv) Submit, where users run the analysis. Users who wish to access documentation can do so by clicking the ‘Help & Privacy’ link located in the top navigation bar.

**Figure 26:**
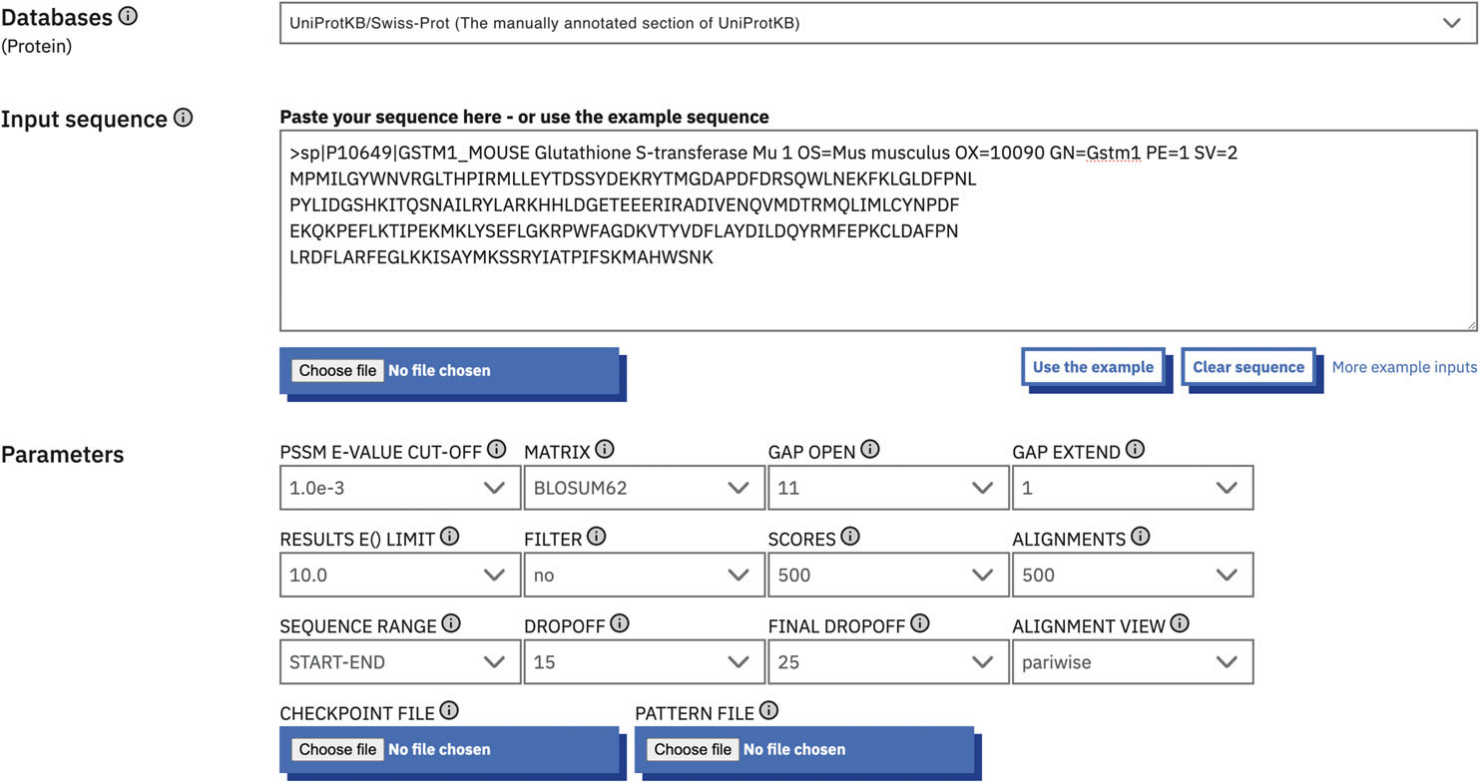
Databases, Input sequence, and Program sections of PSI‐BLAST search.

3 Select the databases to search.
  In the ‘Databases’ section, choose the protein database against which you want to search your query. It is not possible to select multiple databases. For the example depicted here, we choose UniProtKB/Swiss‐Prot, which is the default protein database (Fig. 26).

**Figure 27:**
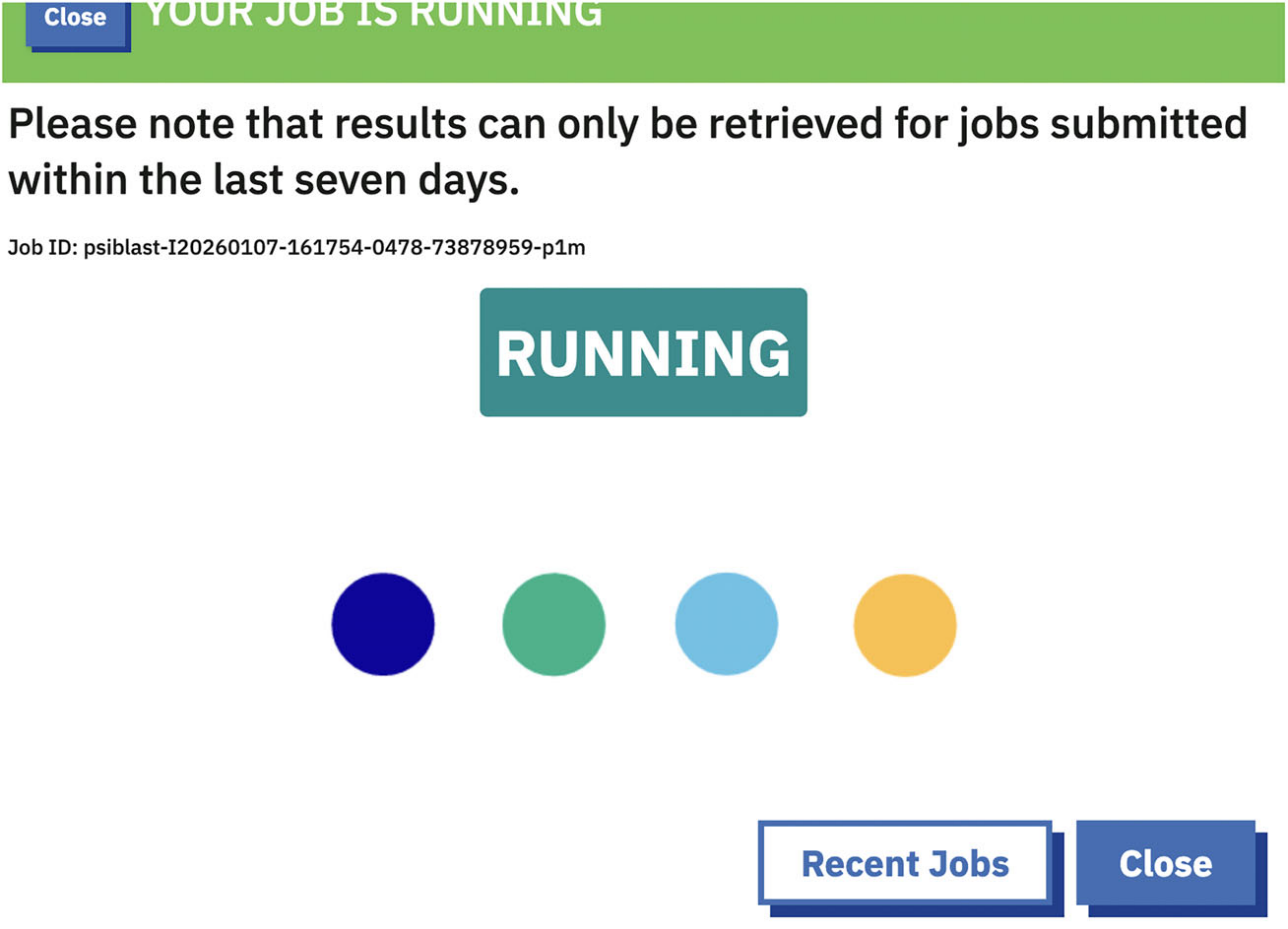
Job ID pop‐up window for PSI‐BLAST search.

4 Enter the input sequence.
  Upload a sequence file (e.g., Fasta, UniProtKB/Swiss‐Prot) using the ‘Choose file’ button. Alternatively, paste the sequence directly into the sequence text box (Fig. 26). You may also use a protein database accession identifier, e.g., UniProtKB:P10649.
5 Set the parameters (Fig. 26).
  Parameters are the settings that control how the search is performed. If you do not change any of these settings, the PSI‐BLAST tool will use its default parameters, which are suitable for most standard searches. Click on the ‘More options’ button to expand the section for the advanced parameters, which include matrix, gap penalties, and E‐value threshold, among others.
  Matrix: Substitution matrix used for scoring (e.g., BLOSUM62, the default for proteins).
  Gap penalties: Gap opening and extension penalties for alignments.
  E‐value threshold: Maximum expected hits to report.
  Adjust the parameter settings to your needs. Tooltips (info icons) provide details about each parameter and are displayed on mouse hover. In this example, we leave the other parameters at their default.
6 Submit the job.
  Once all parameters are set, submit the analysis. Provide an optional Title to briefly describe your job and then click the ‘Submit’ button. A pop‐up window will confirm that your job has been submitted, showing the Job ID and its current status (Fig. 27). The job may be placed in a queue if the servers are busy and will then progress to running and finish, which renders the results page. If any corrections are needed (e.g., missing required fields or invalid parameters), the web form will display warnings (Fig. 6) after you click Submit, prompting you to make the necessary changes.
  You may keep the pop‐up window open to wait for the results or close it to remain on the PSI‐BLAST homepage, to continue with further analysis. You can also navigate to the Recent Jobs page (https://www.ebi.ac.uk/jdispatcher/jobs) to monitor the job progress or return to its results later. This page is particularly useful for long‐running jobs, allowing users to check whether a job has completed or is still in progress. Users can access their submitted jobs on the Recent Jobs page for up to 7 days.

**Figure 28:**
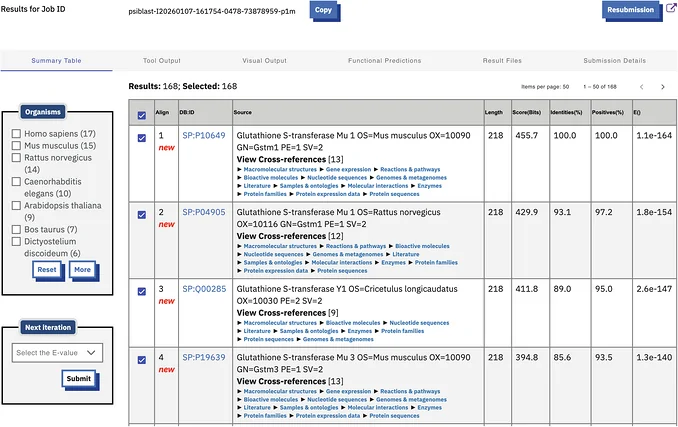
Result Summary Table for PSI‐BLAST search.

**Figure 29:**
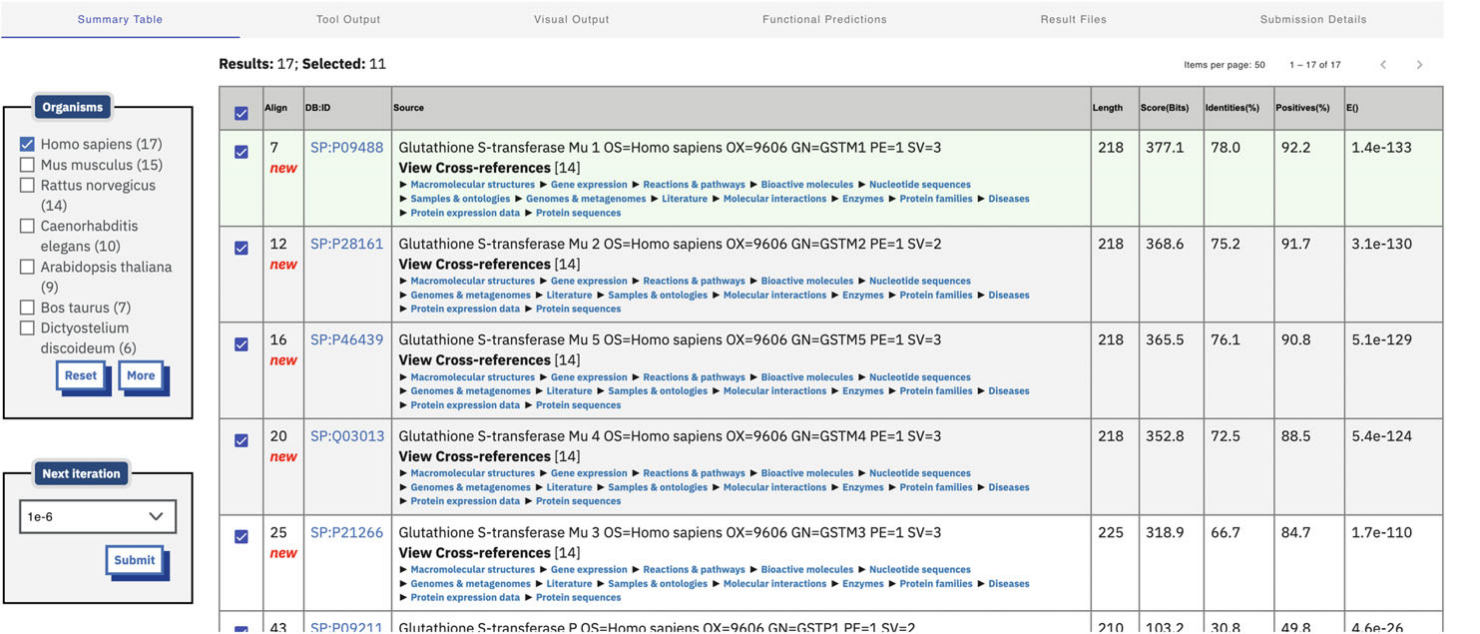
Filter panel for PSI‐BLAST results page for next iteration.

7 View job result summary (Fig. 28).
  After submission, the results page will open automatically when the job finishes. Results will be stored on the servers for only 7 days, so users should download any files they wish to keep for future reference.
  The results interface provides several views that may vary across tools and usually remain consistent within a given tool category. For PSI‐BLAST, the available views include Summary Table, Tool Output, Visual Output, Functional Predictions, Result Files, and Submission Details. The default view displayed is the Summary Table. Use the tabs at the top of the results page to switch between views. The Summary Table lists the top hits returned by the search, including the database, sequence identifier, sequence length, bit score, percentage identity, E‐value, description, and cross‐references. Users can click the sequence identifiers or cross‐reference links to open the corresponding entries in external databases or in EBI Search (Pearce et al., 2025).
  By default, only 50 hits are shown per page; pagination controls allow users to move to the next set of hits.
  A filter panel on the left‐hand side allows users to refine the results using available ‘Organism’ facets, helping to narrow the list of hits to specific organism(s) of interest.
  Selected hits can be used to refine the next iteration by checking the sequence checkboxes. These are included in building the PSSM for the subsequent search (Fig. 29). An inclusion threshold is specified to determine which hits contribute to the PSSM in the next iteration. Newly identified sequences in each iteration are displayed as ‘New’ entries in the Summary Table, allowing users to track the expansion of detected homologs. This iterative selection and refinement process continues for the specified number of iterations, enabling the detection of distant homologs that may not be identified in a single BLASTP search. See step 15 for more details.
  Users can also select individual hits and can view the annotation or alignments of the selected hits with the query sequence; choose ‘Show or hide annotations or alignment.’ An option to download the selected sequences in Fasta format is also available. Additionally, these selected sequences can be directly sent to other MSA tools for further analysis. This supports a seamless workflow from the similarity search to subsequent comparative analyses.

**Figure 30:**
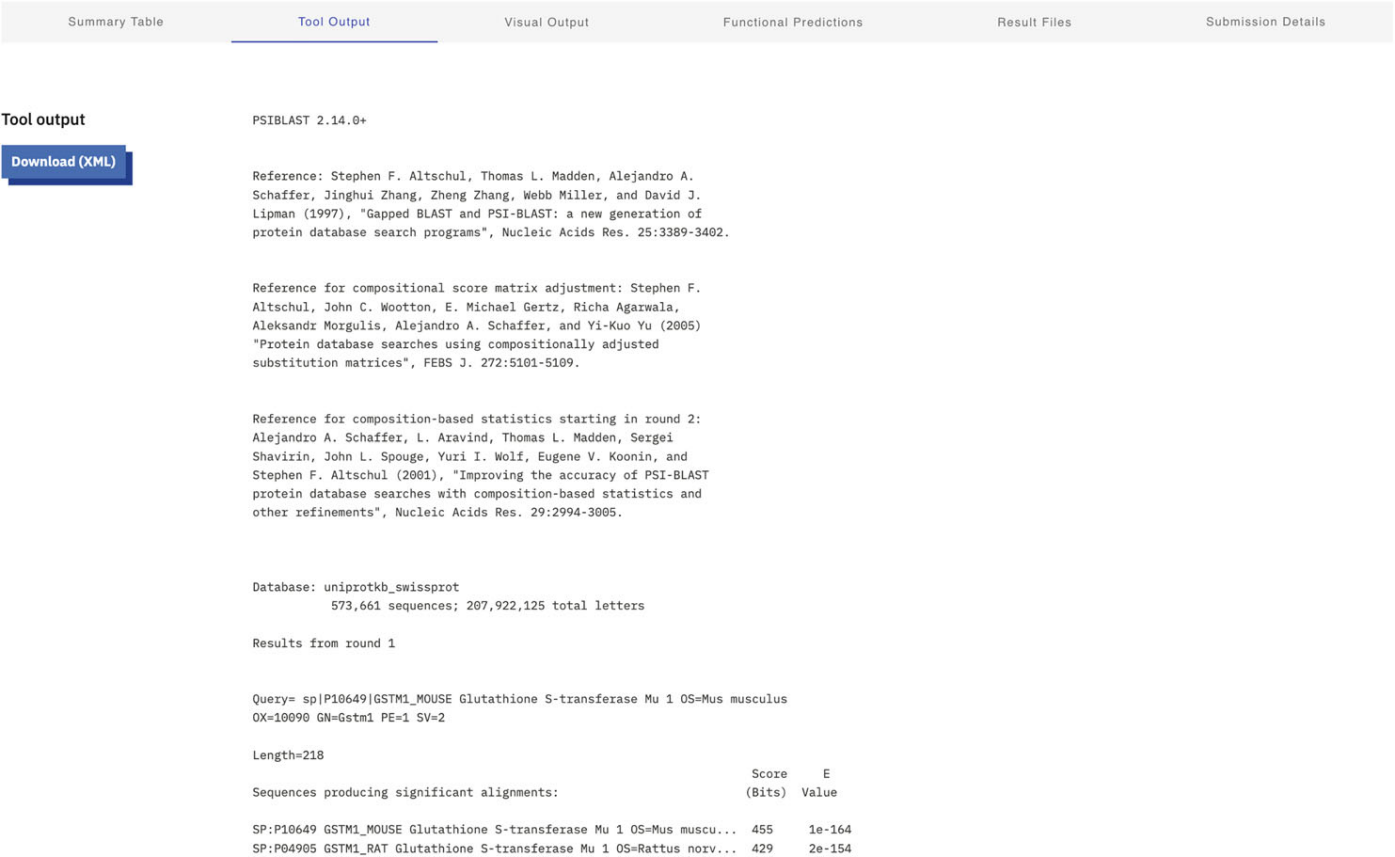
PSI‐BLAST tool raw output.

8 Display the tool's raw output (Fig. 30).
  Click the Tool Output tab to view the raw output produced by the BLAST program. This includes the complete alignment information for each hit as generated by the tool, showing bit scores, E‐values, and the aligned sequences. The raw output represents the original BLAST results without additional formatting or visualization.

**Figure 31:**
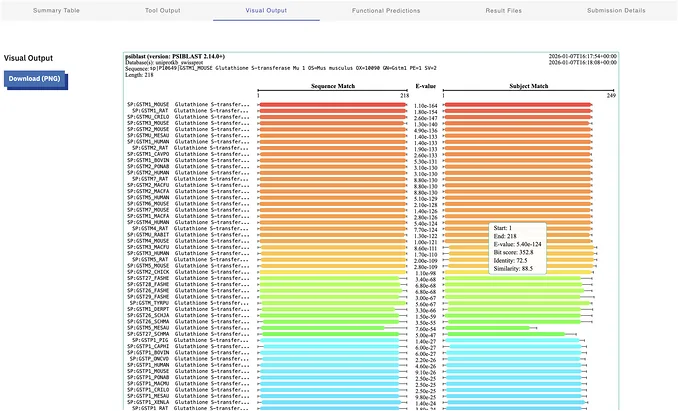
Visual output from PSI‐BLAST search.

9 Visualize the result (Fig. 31).
  The Visual Output (see Current Protocols article: Madeira, Madhusoodanan, Lee, Eusebi, Niewielska, Tivey, Meacham, et al., 2024; Madeira et al., 2025) shows an interactive graphical view of the results, highlighting top hits, alignment coverage, and conserved regions. Sequence bars are clickable to view additional information and link to external resources, and users can adjust the scale, displayed score (e.g., E‐value, bit score, identity), and coloring scheme to explore the alignments. A static image of the visualization can also be downloaded in PNG format for offline use.

**Figure 32:**
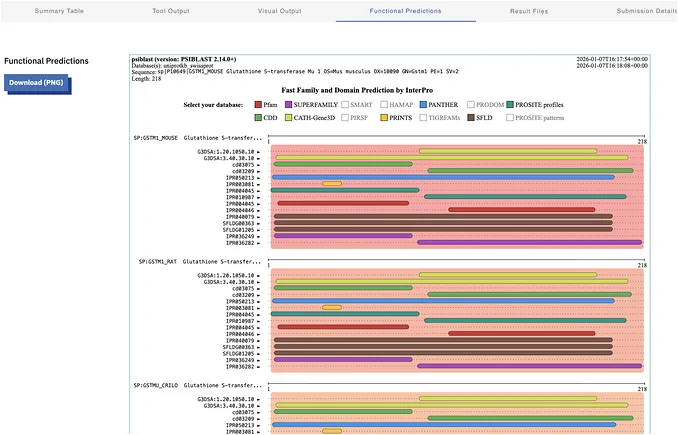
Functional Predictions tab from PSI‐BLAST search.

10 Display functional predictions (Fig. 32).
  The Functional Predictions view (see Current Protocols article: Madeira, Madhusoodanan, Lee, Eusebi, Niewielska, Tivey, Meacham, et al., 2024; Madeira et al., 2025), available under the Functional Predictions tab on the results page, combines the interactive Visual Output with domain annotations. For each hit, predicted domains from InterPro and its member databases (e.g., Pfam, SUPERFAMILY) are displayed as bars overlaid on the alignment. Users can adjust the scale, score type, and coloring scheme as in the Visual Output and toggle individual domain annotations on and off. A static image of the visualization can also be downloaded in PNG format.

**Figure 33:**
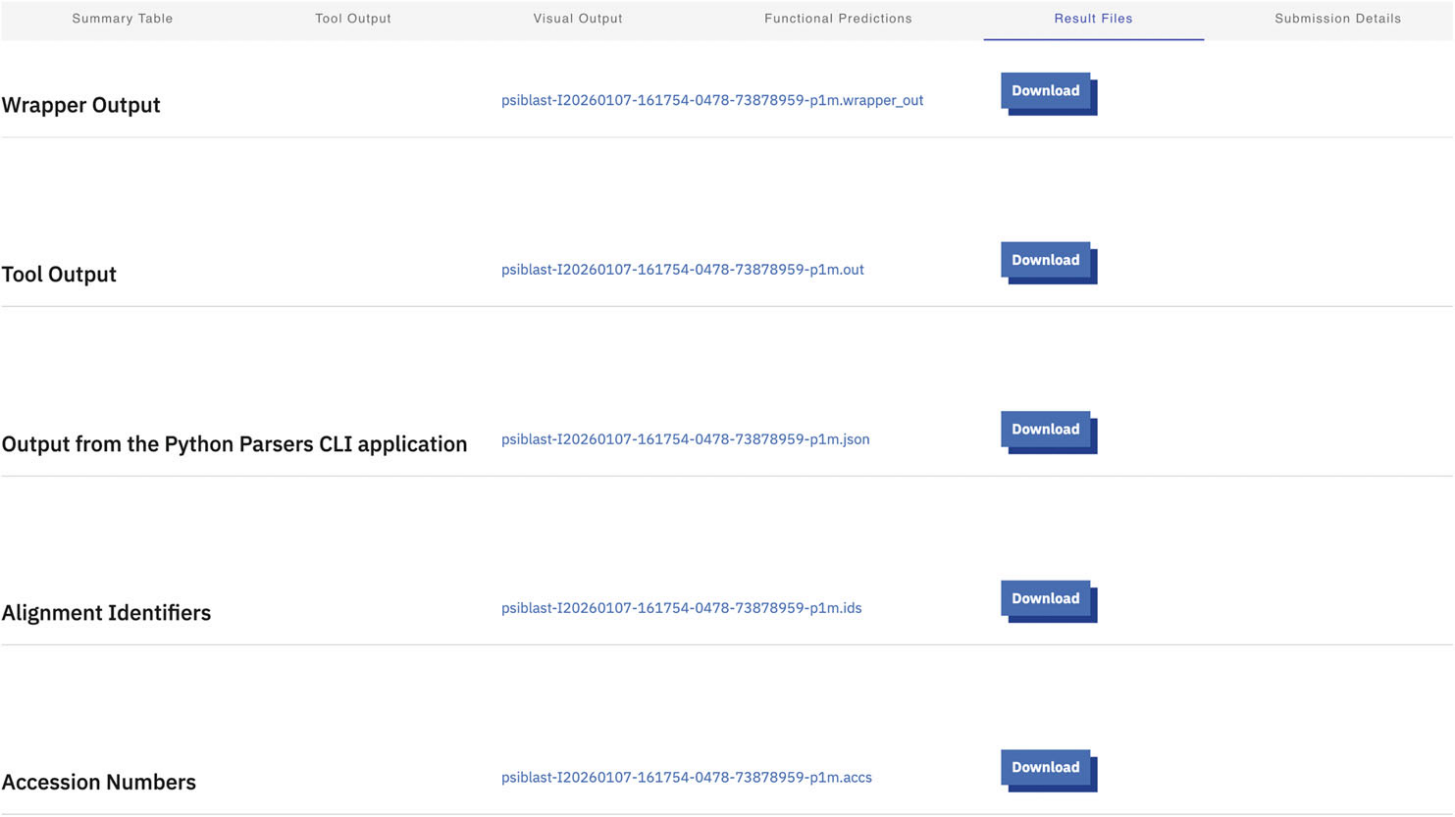
Result Files tab from PSI‐BLAST search.

11 View Result Files.
  The Result Files tab provides access to all files generated by the tool, including the raw alignment output, a list of alignment accessions and identifiers, XML/JSON‐formatted results, PSSM, and other visual outputs. Users can download these files individually or as a single ZIP archive for offline analysis or further processing (Fig. 33).

**Figure 34:**
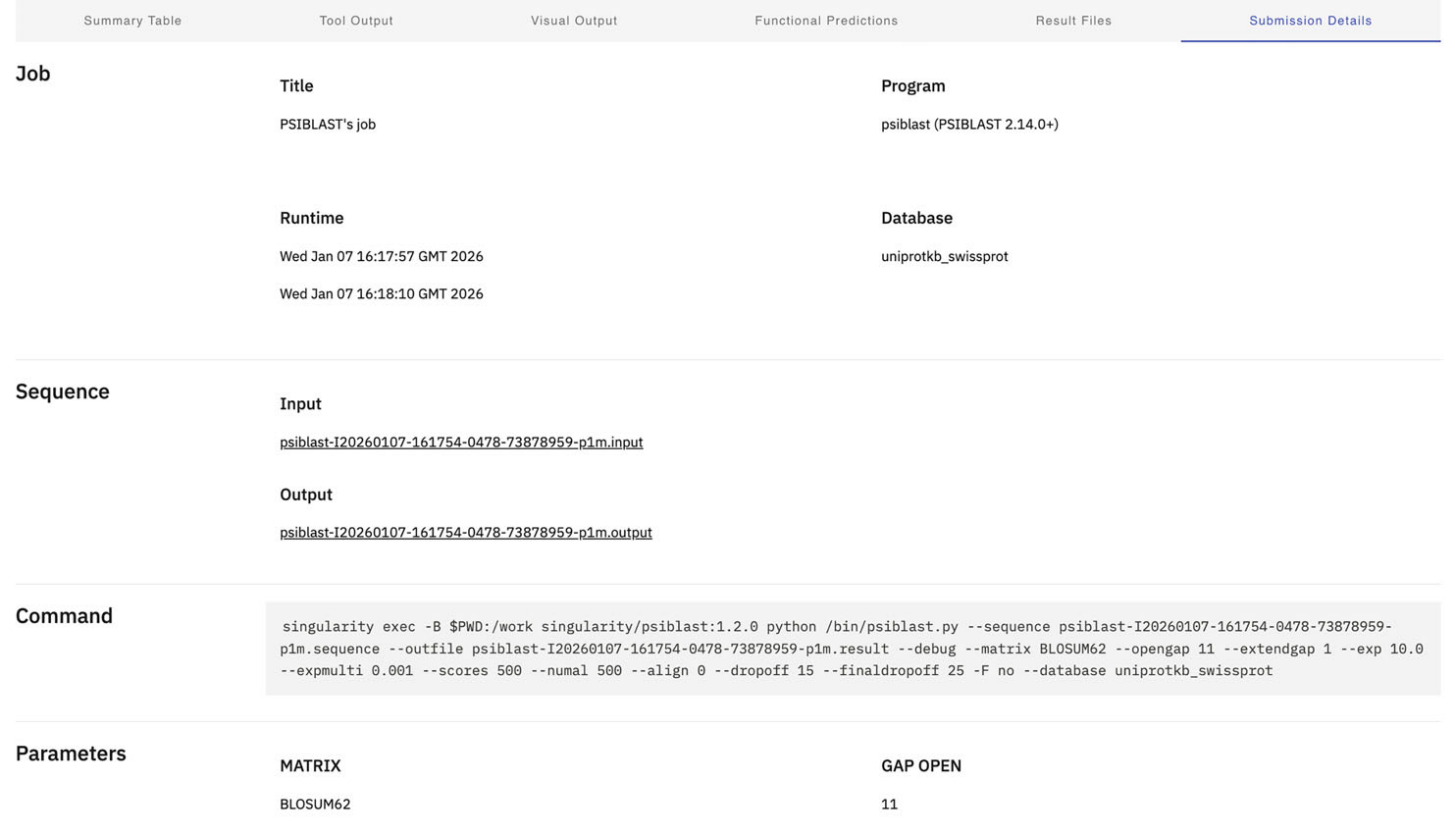
Submission Details tab from PSI‐BLAST search.

12 Display Submission Details (Fig. 34).
  The Submission Details view provides information about the analysis, including the program and its version, selected database, job title, submission date and time, input and output files, command line executed, and input parameter settings. Users can review these details to verify that the submission is correct and determine whether a resubmission is necessary (see step 14).

**Figure 35:**
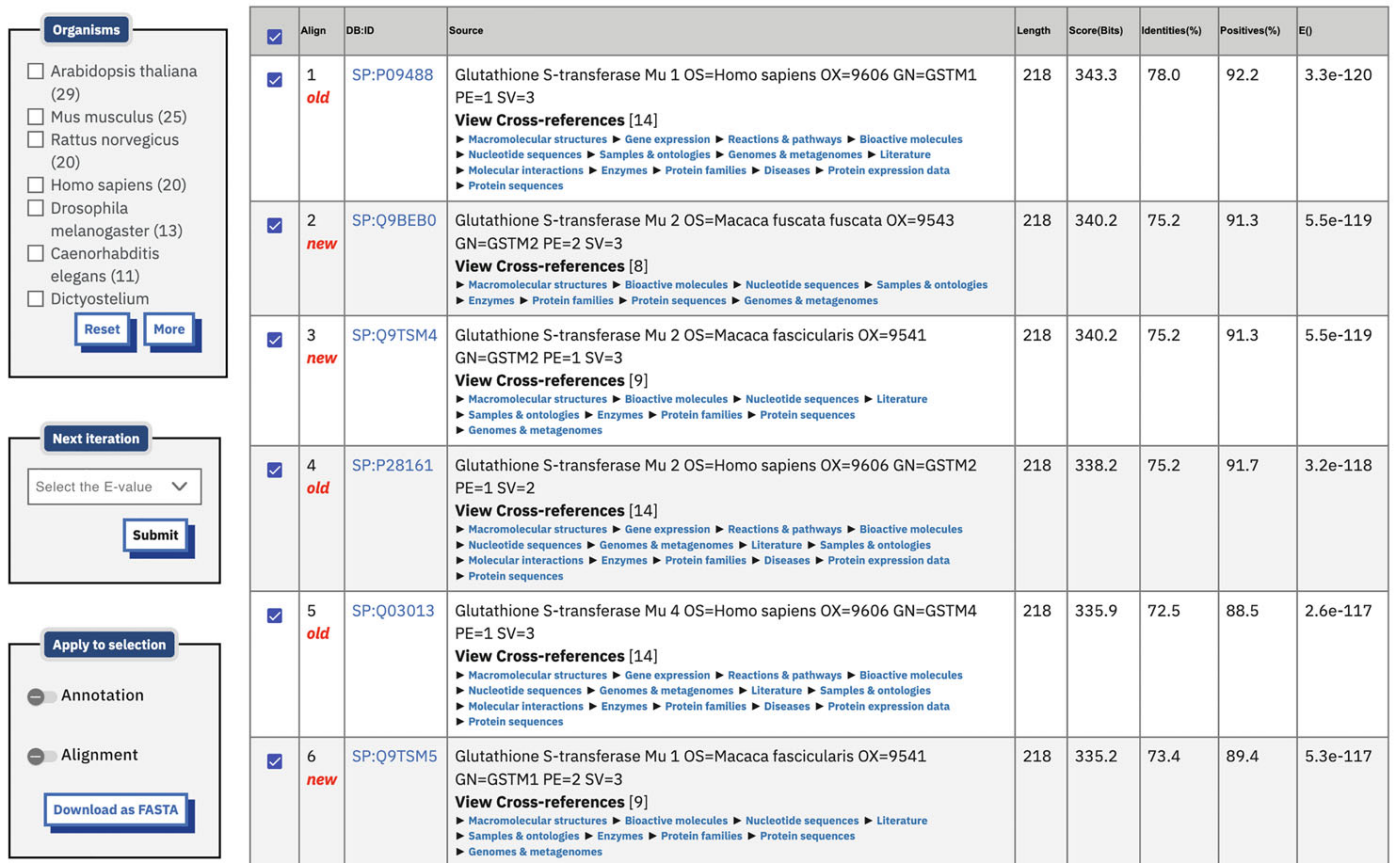
Result Summary Table for the first iteration of PSI‐BLAST search.

13 Resubmit the analysis as needed.
  A new analysis can be prepared by clicking the ‘Resubmission’ button, which starts a new analysis with the current input and settings pre‐filled in the form, allowing you to edit them before submission.
14 Performing iterative search.
  To perform an iterative PSI‐BLAST search, the user first examines the initial BLAST results on the summary page. Sequences that meet the inclusion threshold can be selected by the user and are then used to construct a PSSM for the next iteration. Manual inspection of the hits is recommended to exclude false positives before proceeding.
  In this example, the first PSI‐BLAST iteration was performed from selected sequences with an E‐value ≤ 1e‐6 (Fig. 29). A new Job ID was generated for the iterative search, reflecting the updated PSSM. Subsequent iterations were carried out using this PSSM, allowing the detection of additional homologous sequences. Figure 35 shows the comparison of previously identified hits and newly detected hits after the second iteration.
  PSI‐BLAST builds a PSSM from the MSA of sequences identified in earlier iterations. This matrix captures conserved amino acid patterns at each position in the query sequence, thereby increasing sensitivity and enabling the detection of distant homologs.

## Basic Protocol 4 MULTIPLE SEQUENCE ALIGNMENT USING Clustal Omega

MSA is a technique frequently used for aligning three or more biological sequences to identify conserved regions. These alignments allow researchers to infer homology and investigate the evolutionary relationships among sequences.

Clustal Omega (Sievers & Higgins, 2018; Sievers et al., 2011; also see Current Protocols article: Sievers & Higgins, 2014) is a fast and scalable MSA tool that generates high‐quality alignments using seeded guide trees and hidden Markov model (HMM)‐based profile‐profile comparison techniques.

### Necessary Resources

#### Hardware

Any computer with internet access.

##### Software

A modern web browser (e.g., Chrome, Firefox, Edge, Safari).

##### Input

A plain‐text file containing three or more protein or nucleotide sequences in any supported format (e.g., Fasta, EMBL, GenBank, PHYLIP, UniProtKB/Swiss‐Prot) can be pasted into the form or uploaded.
Alternatively, a database identifier in the format database:identifier.

##### Example for this protocol

A Fasta‐format multiple‐sequence file containing a collection of myosin whey acidic protein (WAP) sequences.
  Fasta‐format sequences: https://www.ebi.ac.uk/Tools/dbfetch/dbfetch?db=uniprotkb&id=wap_rat%2Cwap_mouse%2Cwap_pig&format=fasta&style=raw&Retrieve=Retrieve

**Figure 36:**
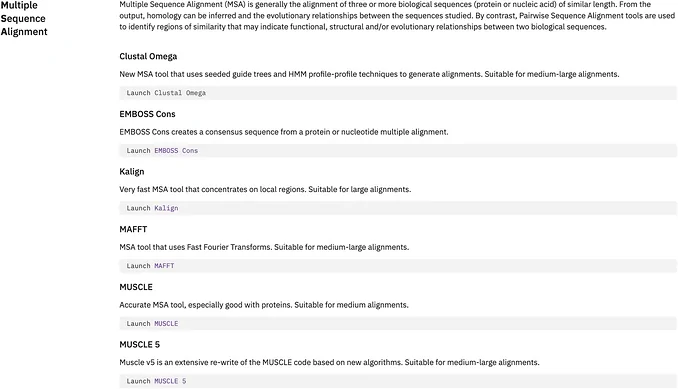
Job Dispatcher Multiple Sequence Alignment tools category page.

1 Open a web browser and go to the MSA page https://www.ebi.ac.uk/jdispatcher/msa using a web browser (Fig. 36).
  This page lists available tools for protein and nucleotide MSA.

**Figure 37:**
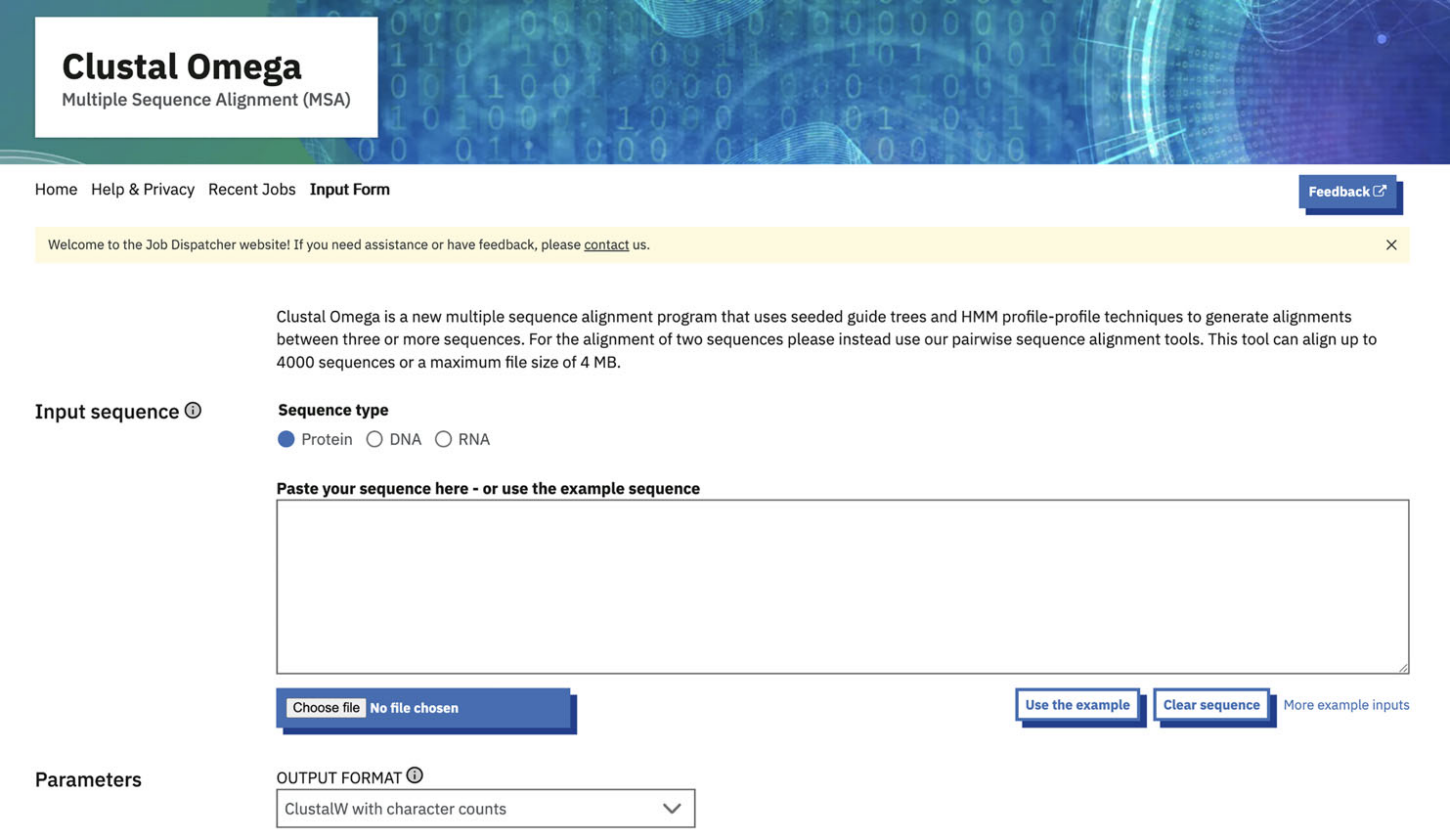
Clustal Omega input form.

2 Click ‘Launch Clustal Omega’ under the Clustal Omega section or directly go to https://www.ebi.ac.uk/jdispatcher/msa/clustalo (Fig. 37).

**Figure 38:**
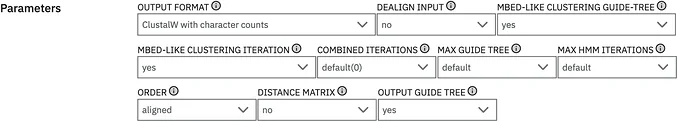
Advanced parameters for Clustal Omega.

3 Select the input type: protein, DNA, or RNA.
4 Enter the input sequence.
  Upload the sequences file (e.g., Fasta, UniProtKB/Swiss‐Prot) using the ‘Choose file’ button. Alternatively, paste the sequences directly into the sequence text box. You may also use a protein database accession identifier.
5 Set the parameters (Fig. 38).
  Parameters are the settings that control how the alignment is performed. If you do not change any of these settings, Clustal Omega will use its default parameters, which are suitable for most standard alignments.
  Click on the ‘More options’ button to access advanced settings, where you can choose the output format (OUTPUT FORMAT), remove existing alignments (DEALIGN INPUT), limit guide‐tree iterations (MAX GUIDE TREE), control HMM iterations (MAX HMM ITERATIONS), set sequence order (ORDER), and adjust several other parameters to fine‐tune the alignment.
  Adjust the parameter settings to your needs. Tooltips (info icons) provide details about each parameter and are displayed on mouse hover. In the example depicted here, we leave the other parameters at their default.

**Figure 39:**
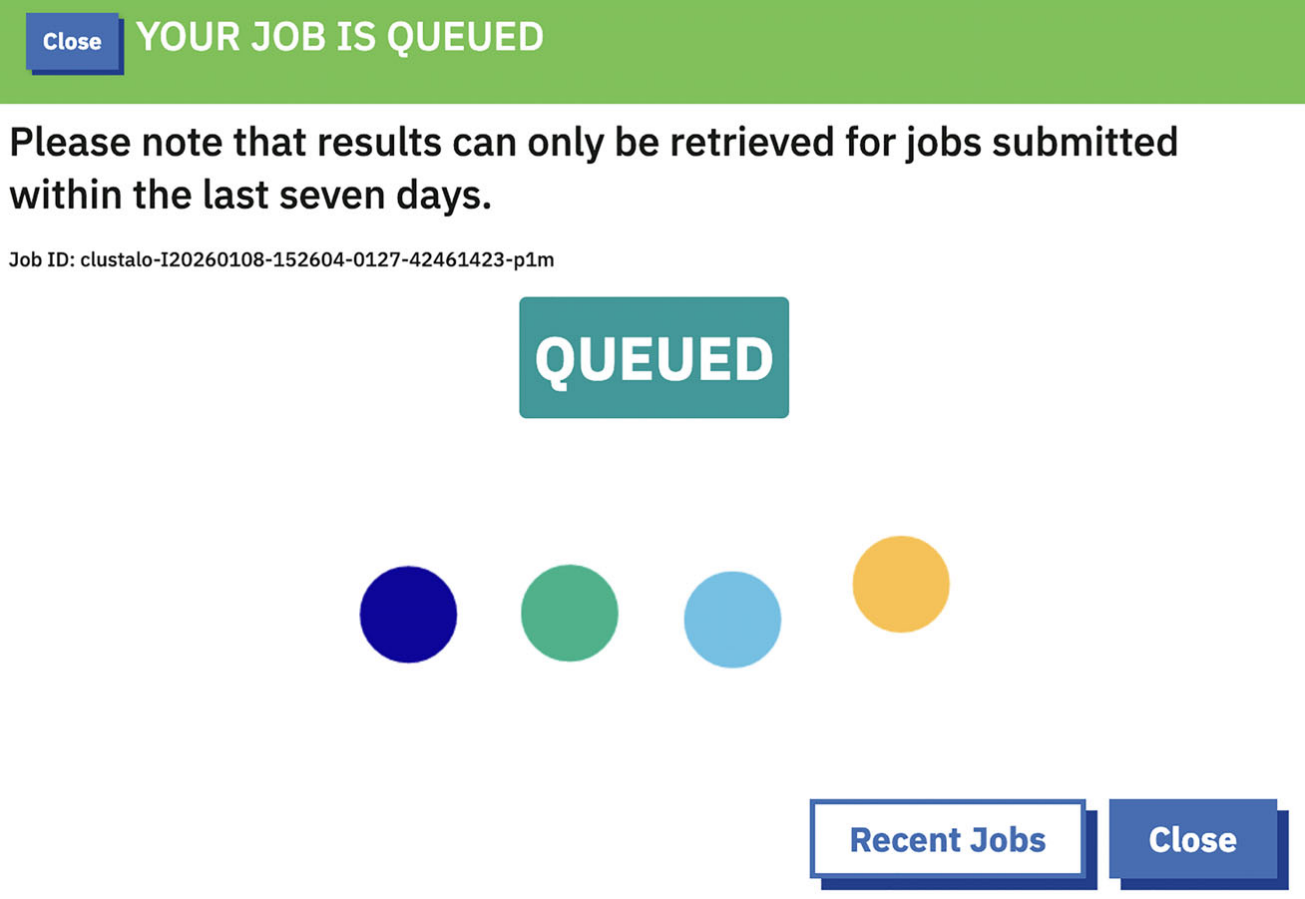
Job ID pop‐up window for Clustal Omega alignment.

**Figure 40:**
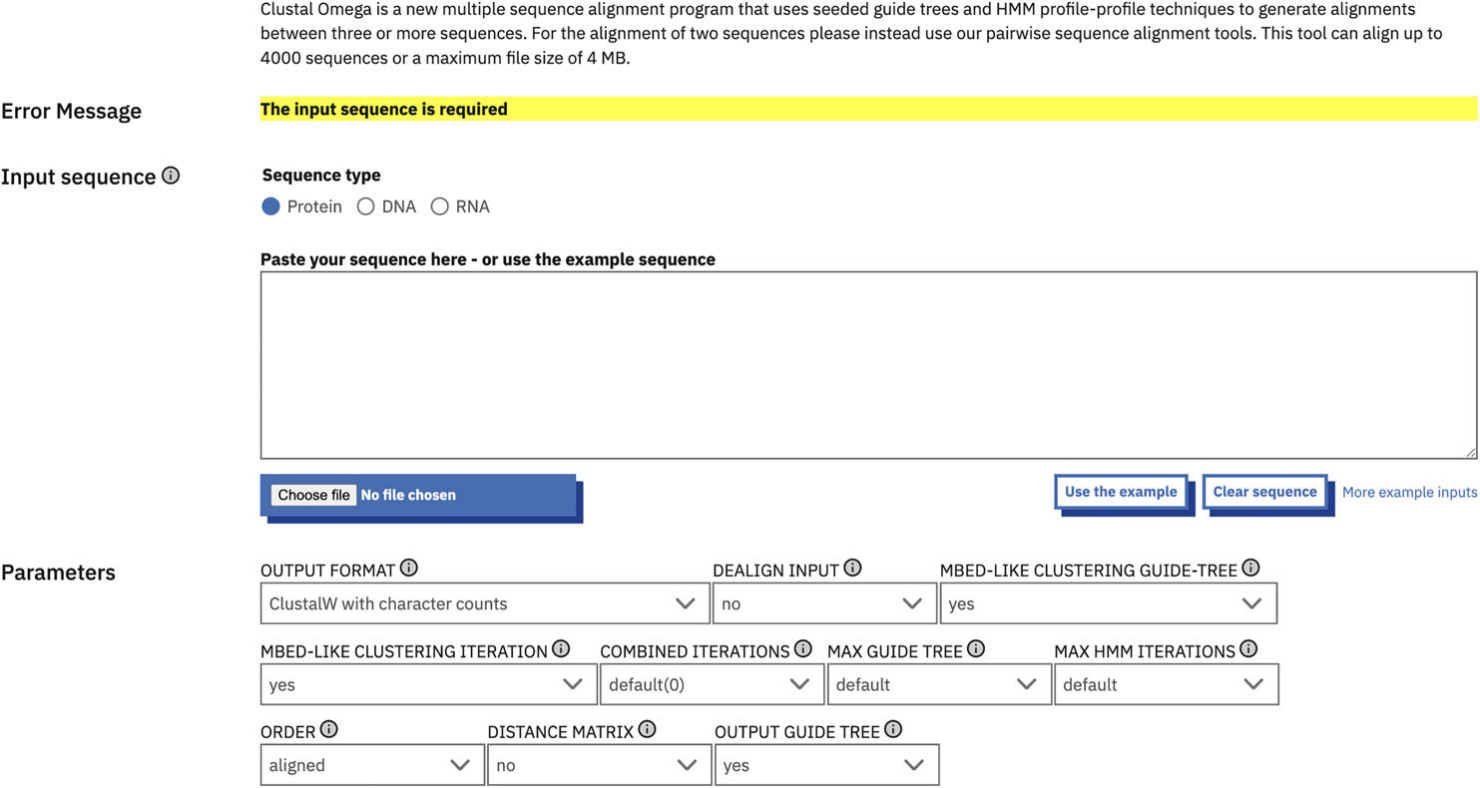
Warnings to provide input sequence for Clustal Omega alignment.

6 Submit the job.
  Once all parameters are set, submit the analysis. Provide an optional Title to briefly describe your job and then click the ‘Submit’ button. A pop‐up window will confirm that your job has been submitted, showing the Job ID and its current status (Fig. 39). The job may be placed in a queue if the servers are busy and will then progress to running and finish, which renders the results page. If any corrections are needed (e.g., missing required fields or invalid parameters), the web form will display warnings (Fig. 40) after you click Submit, prompting you to make the necessary changes.
  You may keep the pop‐up window open to wait for the results or close it to remain on the Clustal Omega homepage to continue with further analysis. You can also navigate to the Recent Jobs page (https://www.ebi.ac.uk/jdispatcher/jobs) to monitor the job progress or return to its results later. This page is particularly useful for long‐running jobs, allowing users to check whether a job has completed or is still in progress. Users can access their submitted jobs on the Recent Jobs page for up to 7 days.

**Figure 41:**
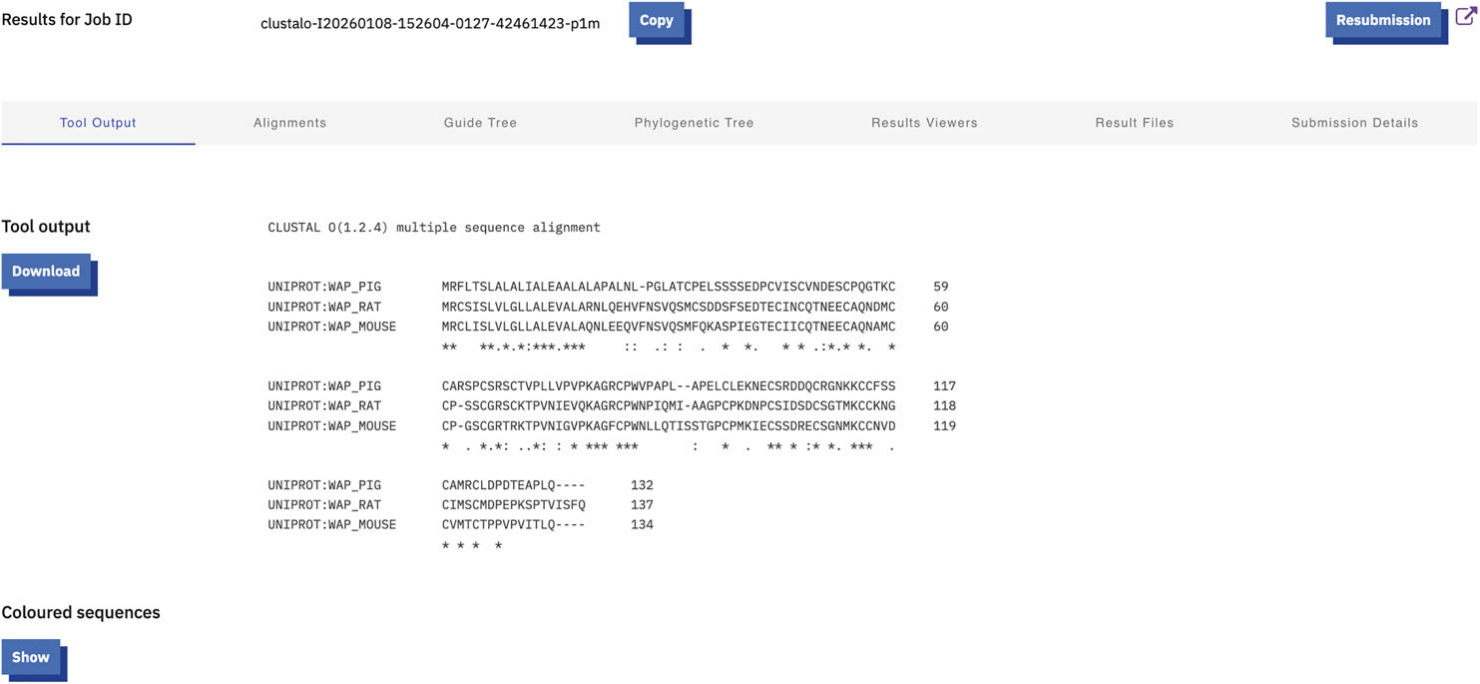
The Tool output tab from Clustal Omega results.

7 View the job results (Fig. 41).
  After submission, the results page will open automatically when the job finishes. Results will be stored on the servers for only 7 days, so users should download any files they wish to keep for future reference.
  The results interface provides several views that may vary across tools but usually remain consistent within a given tool category. For Clustal Omega, the available views include Tool Output, Alignments, Guide Tree, Phylogenetic Tree, Result Viewers, Result Files, and Submission Details. The default view displays the Tool output, showing the alignment produced by Clustal Omega, which can be downloaded from there. Use the tabs at the top of the results page to switch between views.
  In the alignment, gaps are represented by a dash (‘‐‘), ‘*’ denotes fully conserved residues, ‘:’ indicates strongly similar residues, and ‘.’ shows residues with weak similarity. Users can select different color schemes to visualize sequence properties, conservation, or chemical characteristics.
  Color‐coded alignment based on the physicochemical properties of residues appears when you click on ‘Show alignment with colors.’

**Figure 42:**
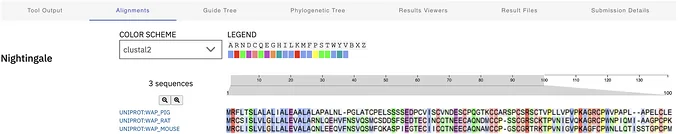
The Alignments tab from Clustal Omega.

8 Display the Alignments page.
  The Alignments tab provides an interactive visualization, powered by Nightingale (Salazar et al., 2023), that allows you to zoom in and out for detailed inspection (Fig. 42). You can also scroll horizontally to see the entire alignment.

**Figure 43:**
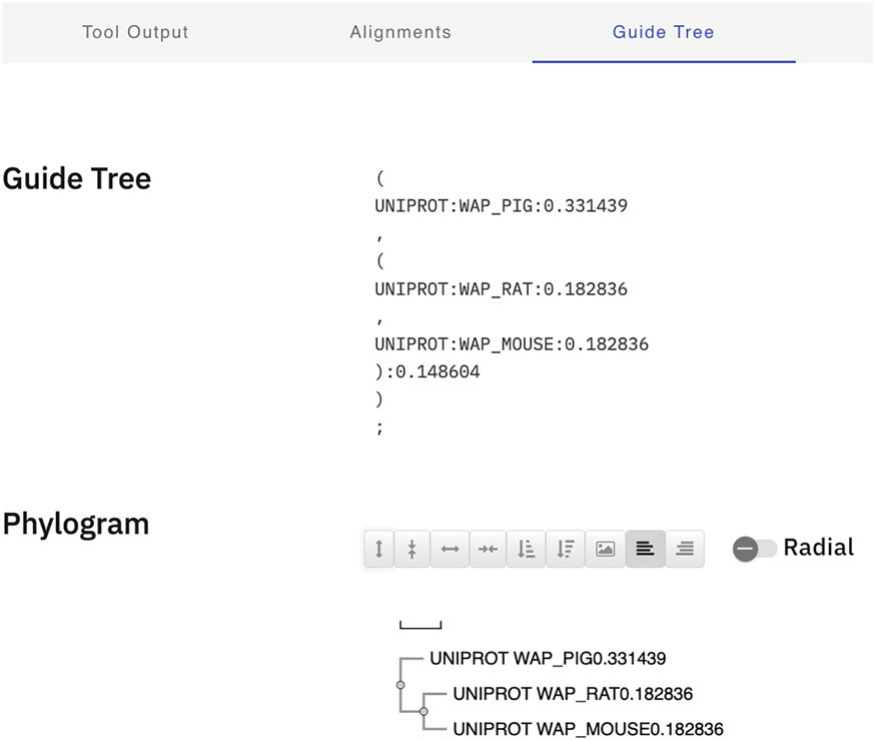
Guide‐tree visualization.

**Figure 44:**
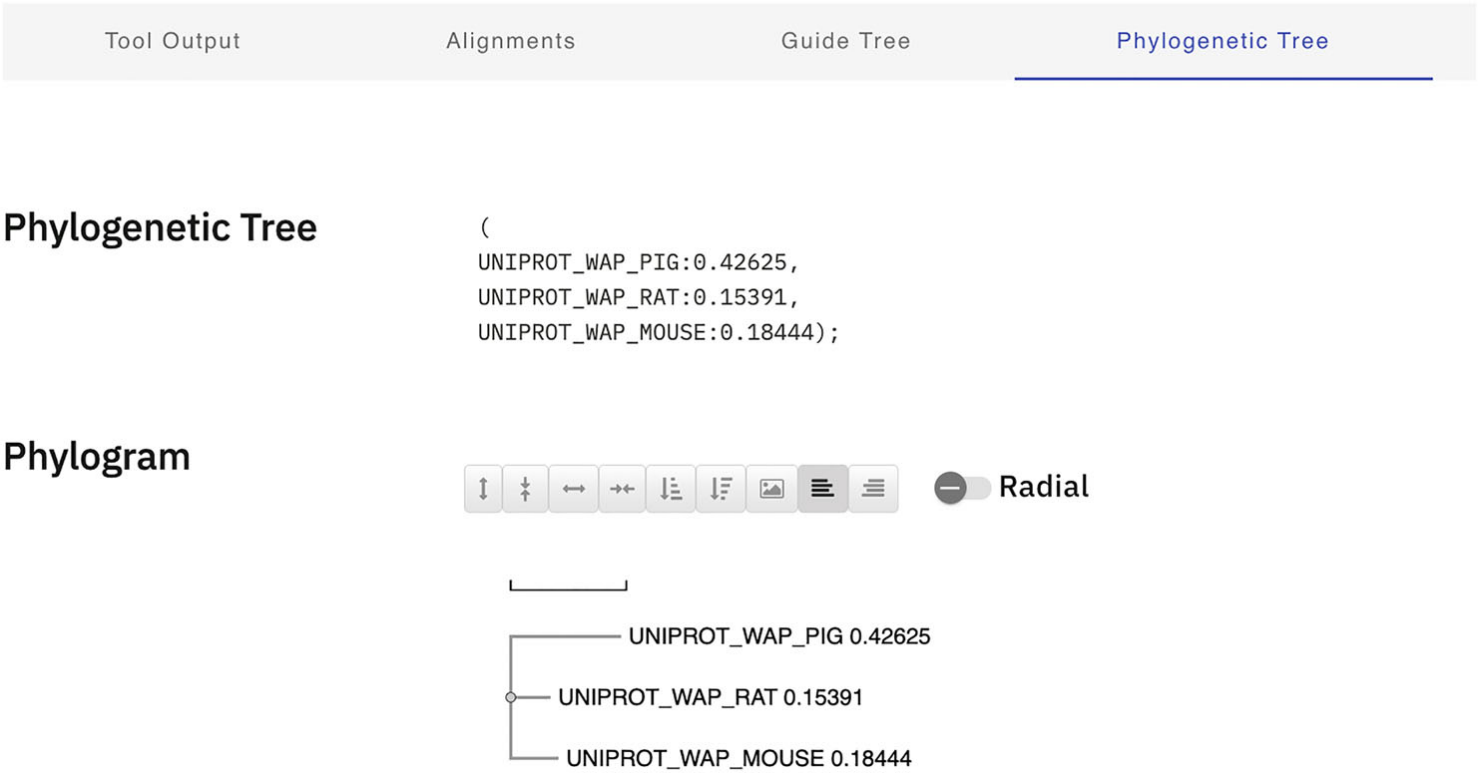
Phylogenetic‐tree visualization.

9 View Guide Tree and Phylogenetic Tree.
  The Guide Tree tab displays the guide tree (Fig. 43), a temporary evolutionary tree used to determine the order in which pairwise sequence alignments are performed.
  The Phylogenetic Tree page (Fig. 44) shows a simple phylogenetic tree calculated from your alignment using the neighbor‐joining method, and the dendrogram visualizations powered by phylotree.js (Shank et al., 2018) are also available.
  Both tree data and visualizations are accessible from the tree pages. The visualization offers several interactive features, such as collapsing branches and re‐rooting.

**Figure 45:**
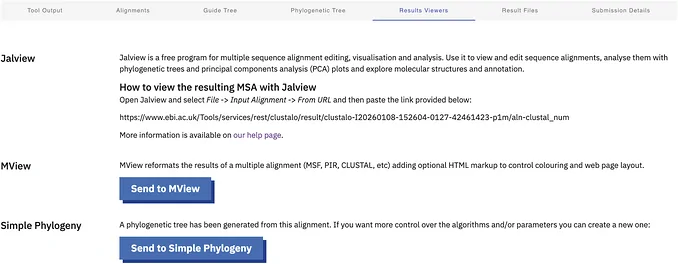
Result Viewers tab from Clustal Omega.

10 View Result Viewers.
  This page enables users to transfer the alignment to other tools. Users can import the alignment directly onto Jalview Desktop (Waterhouse et al., 2009), which offers additional visualization options. It also allows users to send the output alignment directly to Simple Phylogeny and MView (Fig. 45). See Basic Protocol 5 for more information.

**Figure 46:**
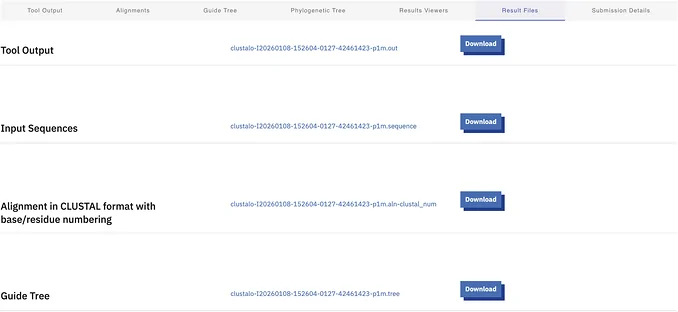
Result Files tab from Clustal Omega.

11 View Result Files.
  This page provides access to all files generated by the tool, including the raw alignment output, Percent Identity Matrix (PIM), guide tree, and phylogenetic tree, among others. Users can download these files individually or as a single ZIP archive for offline analysis or further processing (Fig. 46).

**Figure 47:**
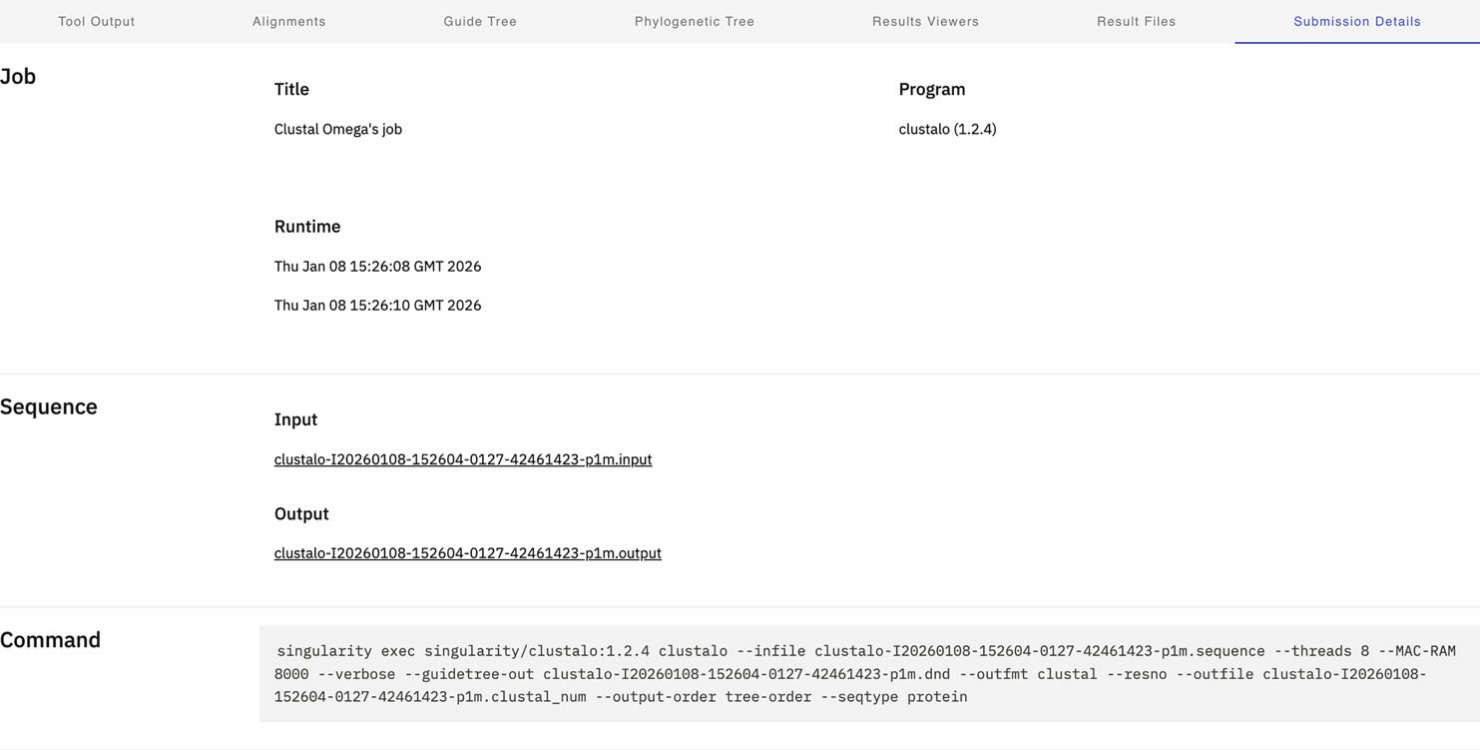
Submission Details tab for Clustal Omega.

12 Display Submission Details (Fig. 47).
  The Submission Details view provides information about the analysis, including the program and its version, job title, submission date and time, input and output files, command line executed, and input parameter settings. Users can review these details to verify that the submission is correct and determine whether a resubmission is necessary (see step 13).

13 Resubmit the analysis as needed.
  A new analysis can be prepared by clicking the ‘Resubmission’ button. This allows you to choose new input sequences or make amendments to parameter values.

## Basic Protocol 5 VISUALIZING MULTIPLE SEQUENCE ALIGNMENTS USING MView

MView (Brown et al., 1998) is a web‐based alignment formatter and visualizer that converts raw sequence alignments into colored, easy‐to‐interpret HTML alignments. It supports outputs from many alignment programs, including Clustal Omega, BLAST, FASTA, and others. MView enhances readability by applying color schemes based on residue conservation and biochemical properties and allows users to export alignments for downstream analysis.

### Necessary Resources

#### Hardware

Any computer with internet access.

##### Software

A modern web browser (e.g., Chrome, Firefox, Edge, Safari).

##### Input

A plain‐text file containing an MSA produced by Clustal Omega or another alignment tool, or BLAST output in any supported format (e.g., Fasta, MSF, BLAST) can be pasted into the form or uploaded.
Alternatively, a Job Dispatcher Job ID from an MSA analysis or from a BLAST search can be used as input.

##### Example for this protocol

A Clustal Omega alignment of myosin WAP sequences (generated in Basic Protocol 4).

**Figure 48:**
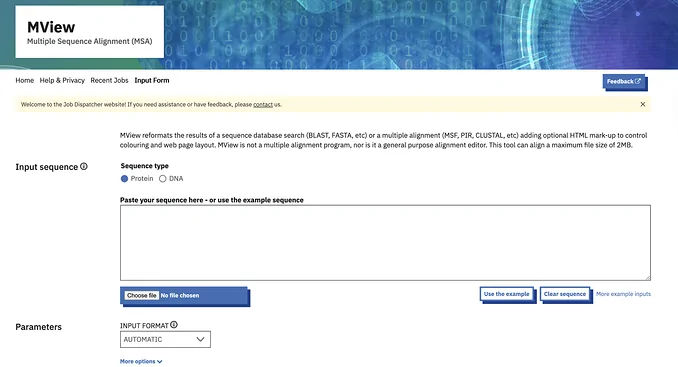
MView input form.

1 Navigate to the MView page: https://www.ebi.ac.uk/jdispatcher/msa/mview.
  This page provides the MView submission form along with the accepted input types and parameter options (Fig. 48).

**Figure 49:**
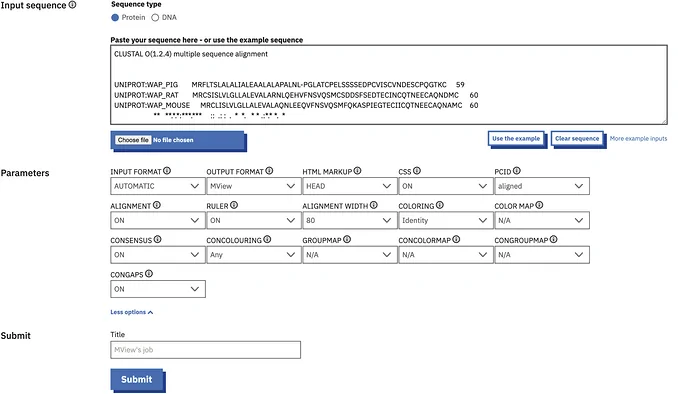
Advanced parameters for MView.

2 Select the sequence type (protein or DNA) and then enter the input sequence.
  Paste the sequence alignment directly into the sequence text box (Fig. 49). Alternatively, upload the alignment file using the ‘Choose file’ button.
  If launching MView directly from a Clustal Omega results page (via Result Viewers), the alignment will transfer automatically, and this step is skipped.

**Figure 50:**
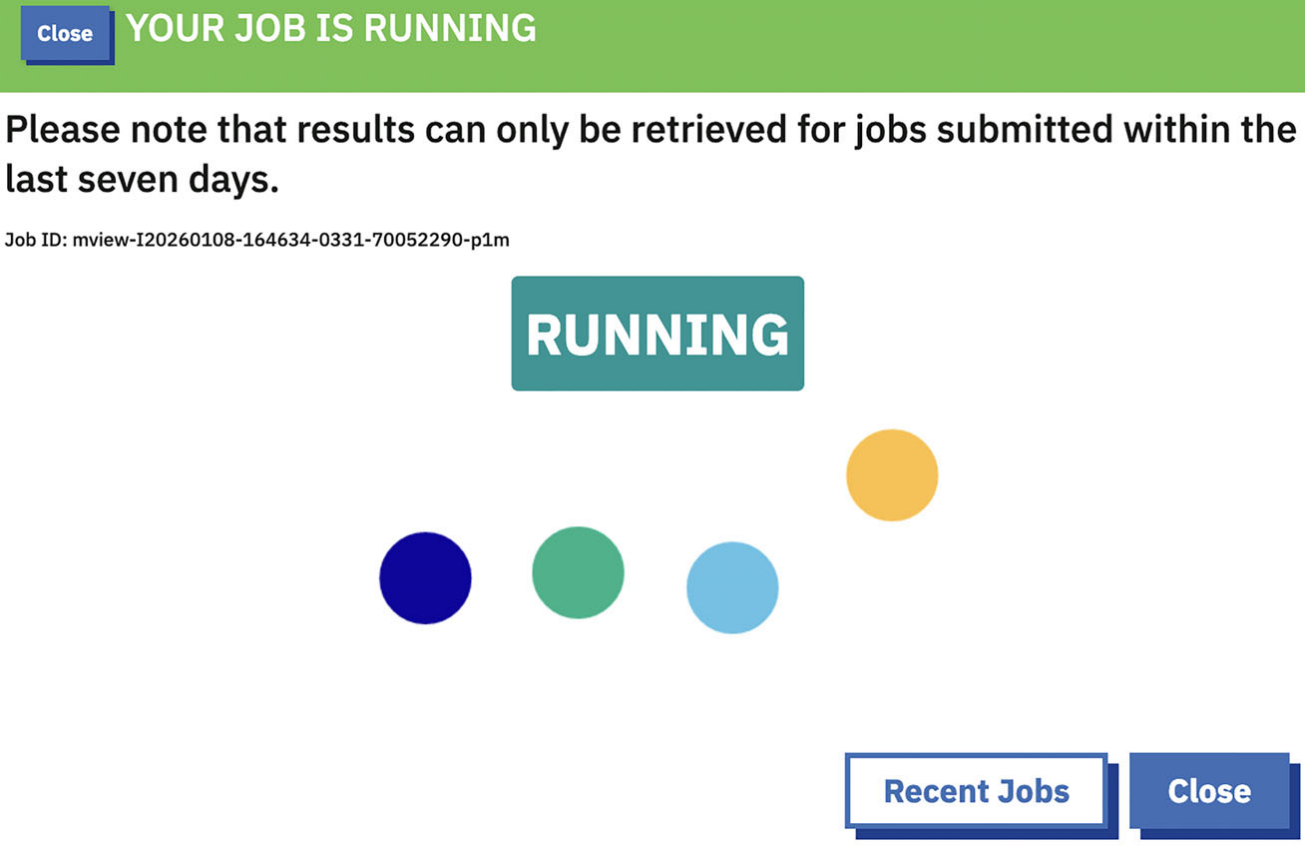
Job ID pop‐up window for MView.

3 Set the parameters (Fig. 49).
  Parameters are the settings that control how the alignment is performed. If you do not change any of these settings, MView will use its default parameters. Click the ‘More options’ button to expand the section for advanced parameters, which include output format and coloring styles, among others. Adjust the parameter settings to your needs. Tooltips (info icons) provide details about each parameter and are displayed on mouse hover. In the example depicted here, we leave the other parameters at their default.
4 Submit the job.
  Once all parameters are set, submit the analysis. Provide an optional Title to briefly describe your job and then click the ‘Submit’ button. A pop‐up window will confirm that your job has been submitted, showing the Job ID and its current status (Fig. 50). The job may be placed in a queue if the servers are busy and will then progress to running and finish, which renders the results page. If any corrections are needed (e.g., missing required fields or invalid parameters), the web form will display warnings after you click Submit, prompting you to make the necessary changes.
  You may keep the pop‐up window open to wait for the results or close it to remain on the MView homepage to continue with further analysis. You can also navigate to the Recent Jobs page (https://www.ebi.ac.uk/jdispatcher/jobs) to monitor the job progress or return to its results later. This page is particularly useful for long‐running jobs, allowing users to check whether a job has completed or is still in progress. Users can access their submitted jobs on the Recent Jobs page for up to 7 days.

**Figure 51:**
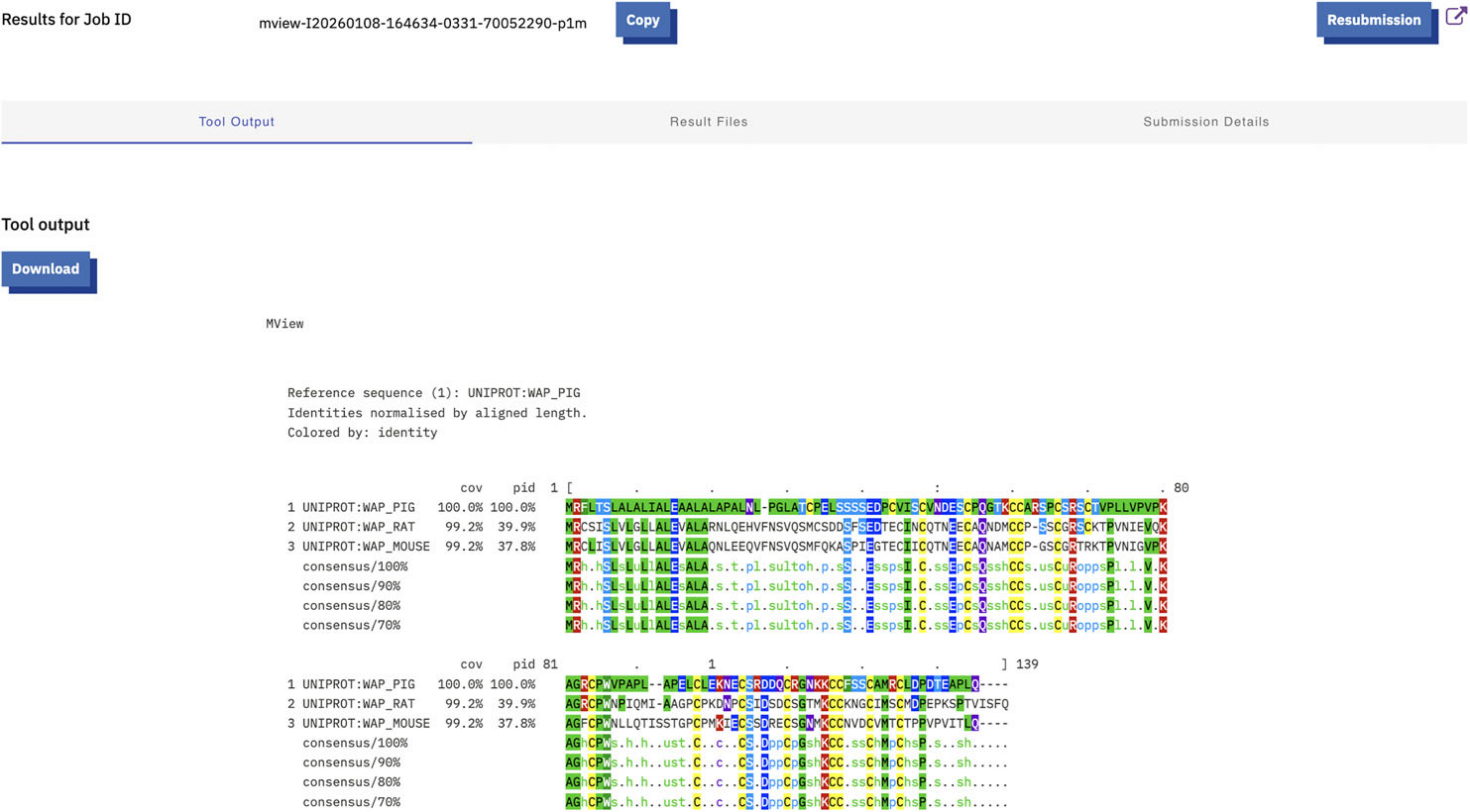
Tool output tab from MView results.

5 View the job results (Fig. 51).
  After submission, the results page will open automatically when the job finishes. Results will be stored on the servers for only 7 days, so users should download any files they wish to keep for future reference.
  The MView results interface includes the following tabs: Tool Output, Result Files, and Submission Details. The default view displays the Tool output, showing the formatted alignment produced by MView, which can be downloaded from there as an HTML file. The MView output shows how similar each sequence is to the reference and highlights where the amino acids match or differ across the alignment. Positions that are conserved appear the same across sequences, whereas variable positions show differences. The consensus lines underneath summarize the most common residue type at each position, giving a quick visual indication of which regions are highly conserved and which are more variable.

**Figure 52:**
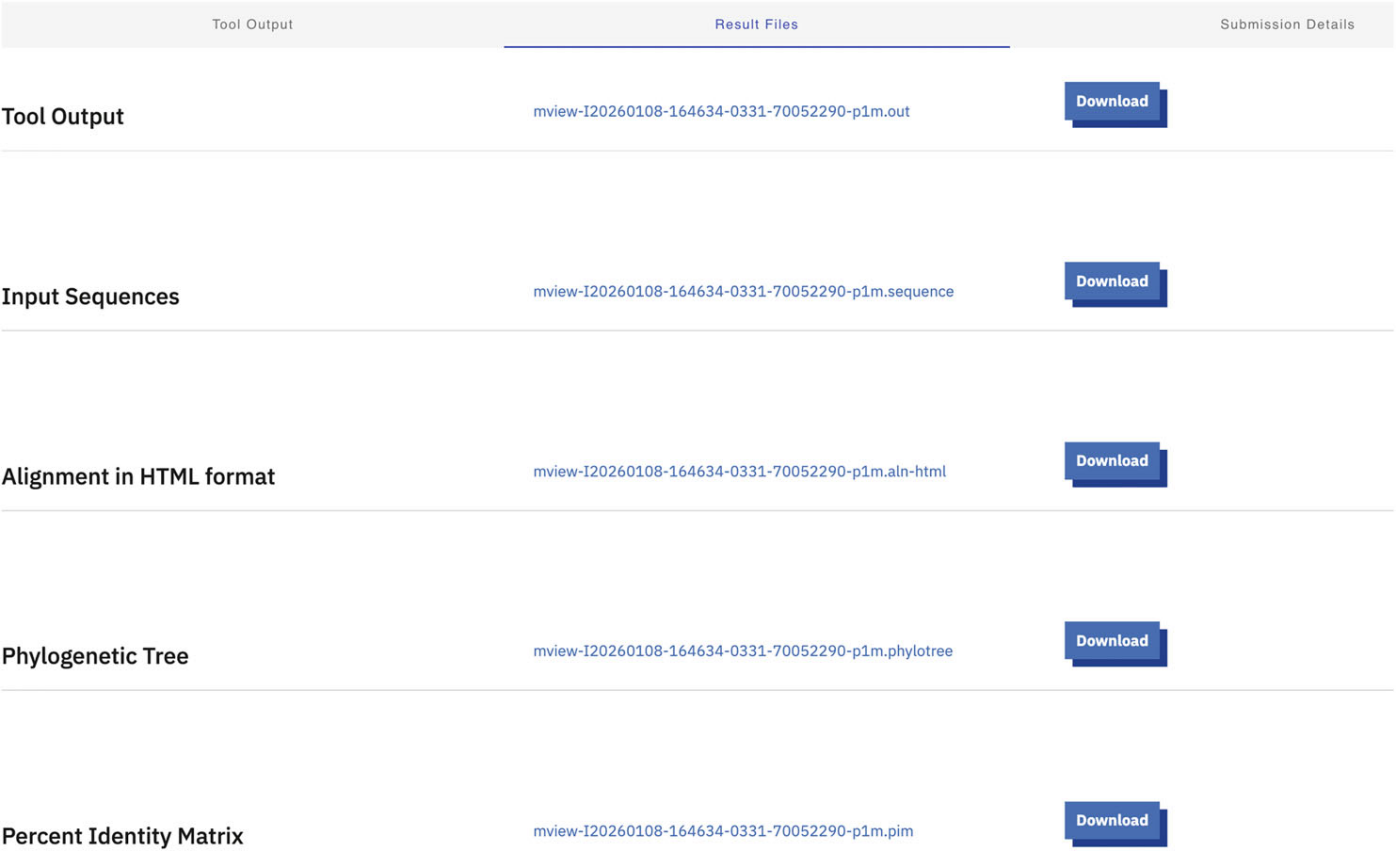
Result Files tab from MView.

6 View Result Files.
  The Result Files tab provides access to all files generated by the tool, including the raw alignment output and PIM, among others. Users can download these files individually or as a single ZIP archive for offline analysis or further processing (Fig. 52).

**Figure 53:**
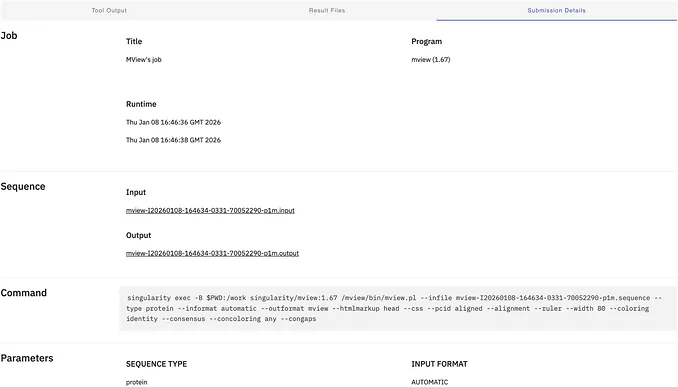
Submission Details tab for MView.

7 Display Submission Details (Fig. 53).
  The Submission Details view provides information about the analysis, including the program and its version, job title, submission date and time, input and output files, command line executed, and input parameter settings. Users can review these details to verify that the submission is correct and determine whether a resubmission is necessary (see step 8).

8 Resubmit the analysis as needed.
  A new analysis can be prepared by clicking the ‘Resubmission’ button. This allows you to choose a new input or make amendments to parameter values.

## Basic Protocol 6 WORKFLOW FROM BLAST RESULTS TO Clustal Omega TO MView

This protocol describes a complete analysis workflow that begins with an SSS (performed by BLAST, described in Basic Protocol 2), continues through MSA with Clustal Omega (Basic Protocol 4), and concludes with alignment visualization using MView (Basic Protocol 5). The workflow is suitable for identifying homologous sequences, generating high‐quality alignments, and producing publication‐ready visualizations.

### Necessary Resources

#### Hardware

Any computer with internet access.

##### Software

A modern web browser (e.g., Chrome, Firefox, Edge, Safari).

##### Input

A nucleotide or protein sequence in a supported format (e.g., Fasta, EMBL, GenBank, PHYLIP, UniProtKB/Swiss‐Prot) can be pasted into the form or uploaded.
Alternatively, a database identifier in the format database:identifier (e.g., UniProtKB:GSTM1_MOUSE).

##### Example for this protocol

The mouse protein Glutathione S‐transferase Mu 1 (UniProtKB:P10649) will be used as input:
  Entry details: https://www.uniprot.org/uniprotkb/P10649/entry
  Fasta‐format sequence: https://rest.uniprot.org/uniprotkb/P10649.fasta

**Figure 54:**
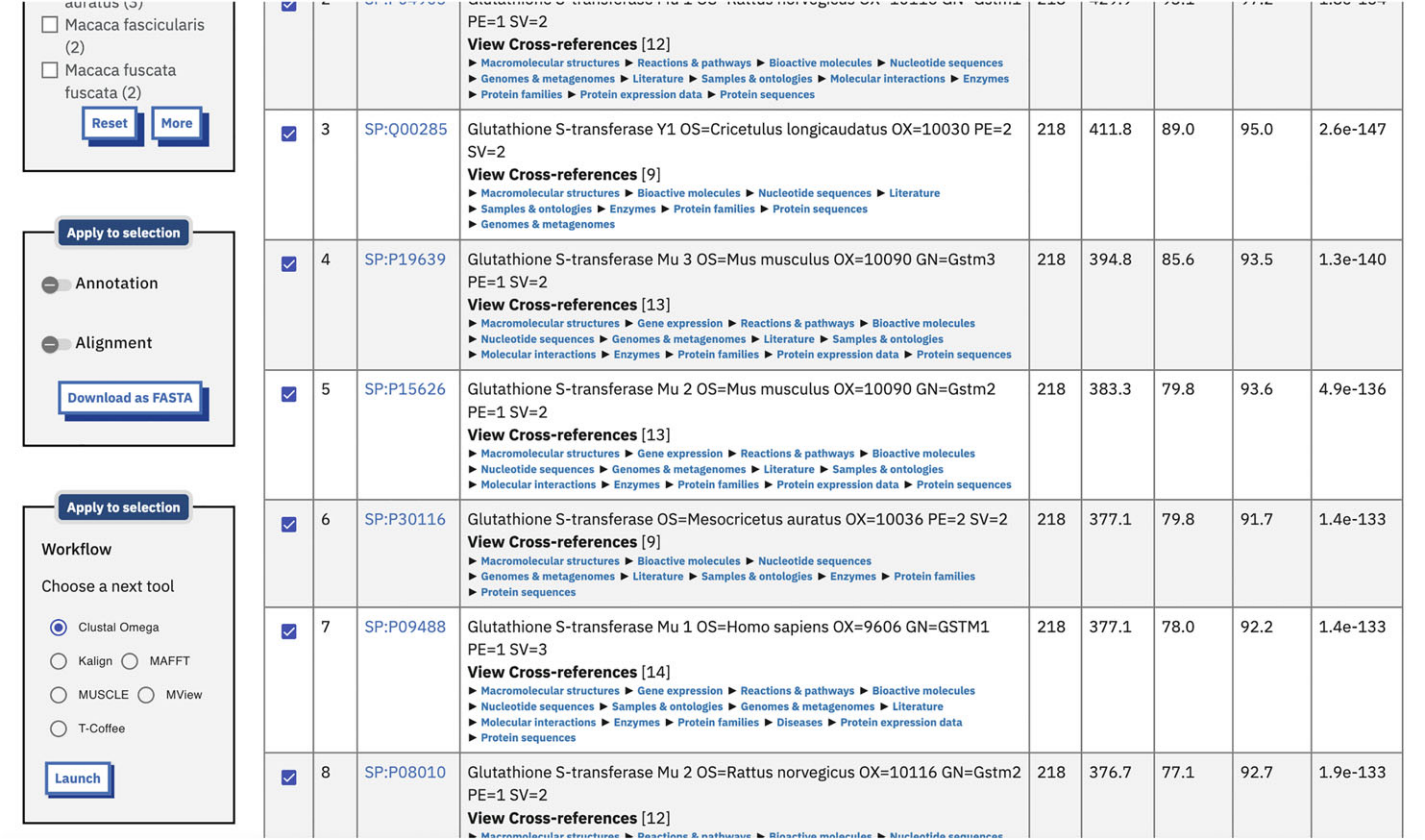
Clustal launched on selected hits from the BLAST results page.

1 Identify homologous sequences using BLAST (see Basic Protocol 2 for details; only the workflow connection is described here).
  Submit your query sequence to BLASTP (https://www.ebi.ac.uk/jdispatcher/sss/ncbiblast) using the default parameters. Inspect the hit list from the result Summary Table page and select sequences of interest by selecting the hits, typically homologs across species or proteins within a family.
  Launch ‘Clustal Omega’ on the selected hits from the Workflow section on the left panel of the results page (Fig. 54).

**Figure 55:**
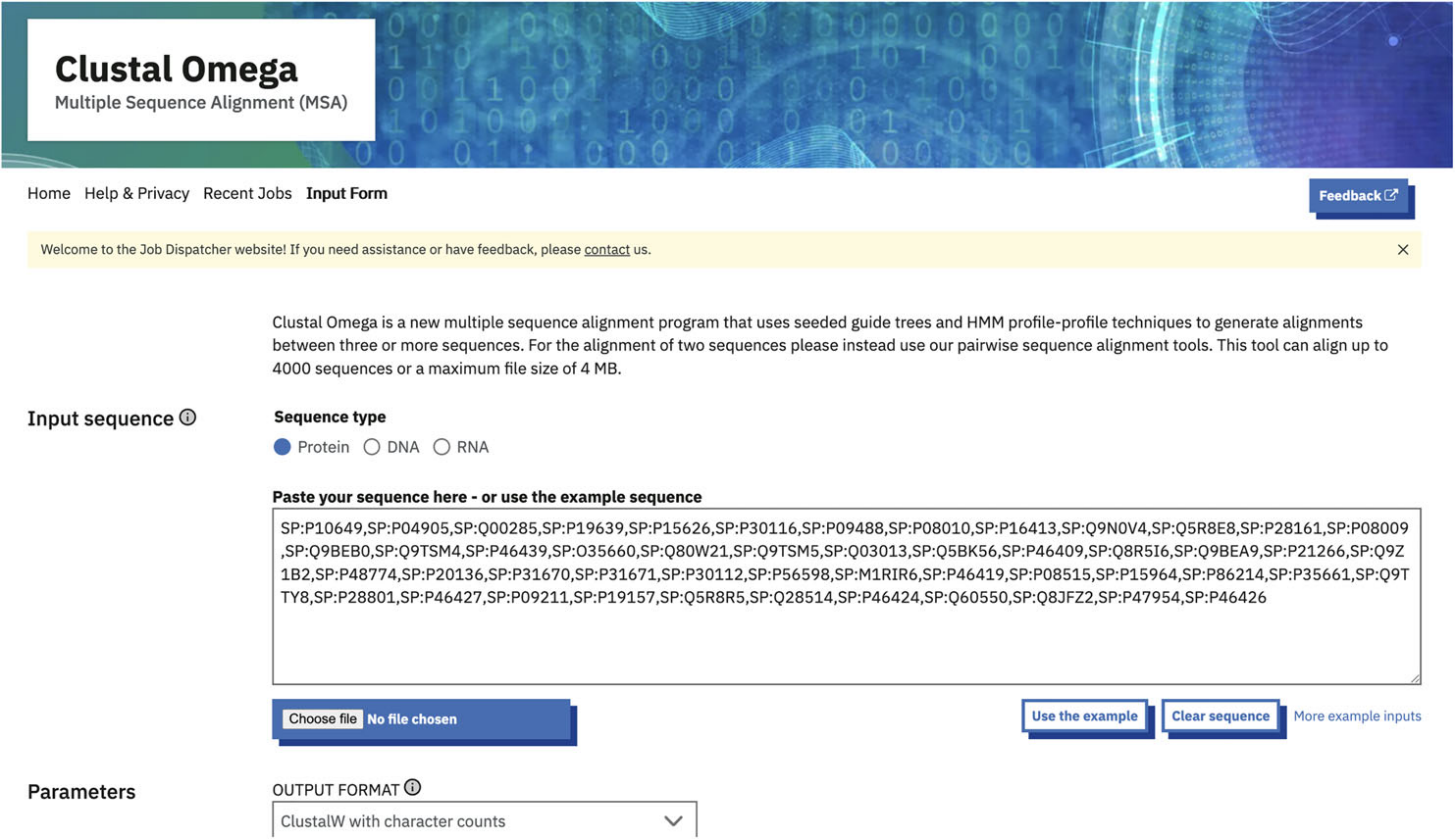
Clustal Omega input form with already selected sequences in database:identifier format.

**Figure 56:**
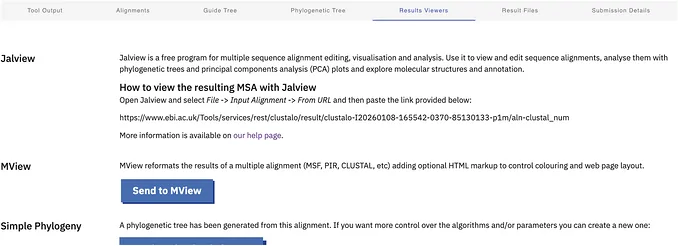
Send the Clustal result to MView from the Results Viewers tab in Clustal Omega.

2 Generate an MSA with Clustal Omega (see Basic Protocol 4 for a complete description of the procedure).
  Once you click ‘Launch,’ it navigates to the Clustal Omega web page, with already‐populated sequences in the database:identifier format (Fig. 55). Leave parameters at default unless special settings are required, submit the job, and wait for the alignment to finish.
  From the results page of Clustal Omega, navigate to the Result Viewers tab and click on the ‘Send to MView’ button (Fig. 56).

**Figure 57:**
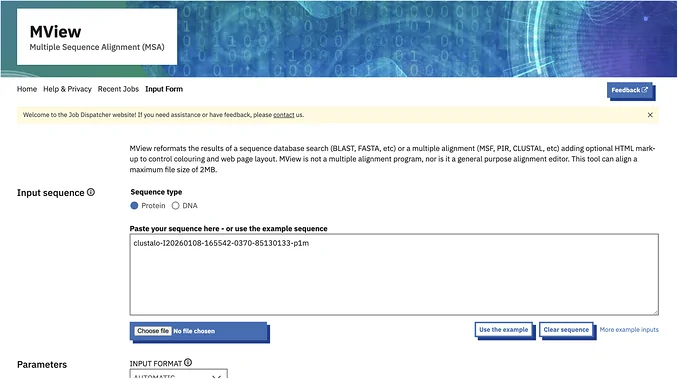
Job ID pop‐up window for Clustal Omega alignment.

**Figure 58:**
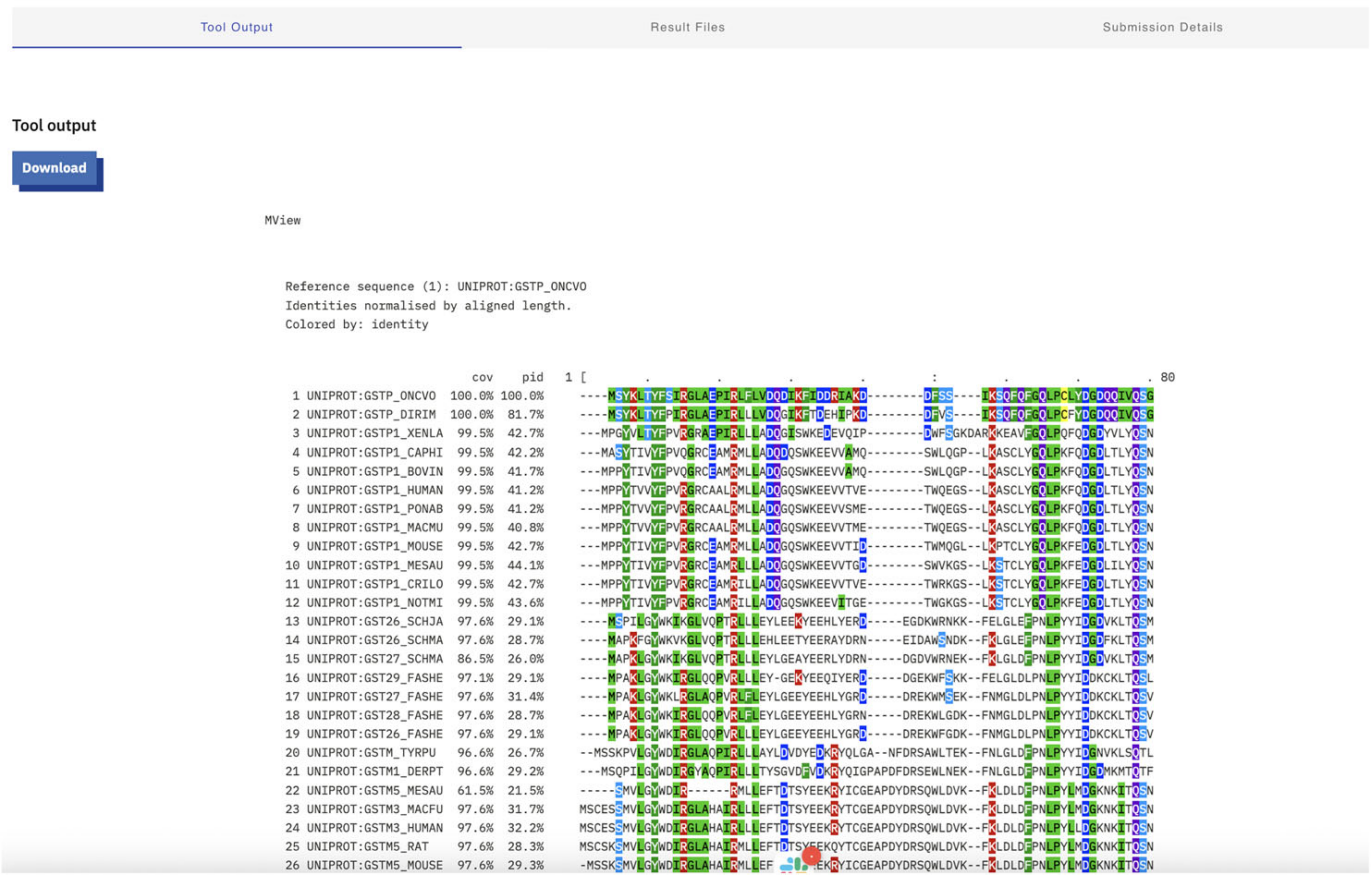
MView results page for the selected Blast hits.

3 Visualize and format the alignment with MView (see Basic Protocol 5 for details).
  When the alignment is sent from Clustal Omega to MView, MView automatically retrieves the alignment using the previous Clustal Omega Job ID (Fig. 57), so the alignment data do not need to be re‐entered manually. You can either leave parameters at their default or choose special settings if required.
  Once the job is finished, you can view, color, and export the formatted alignment (Fig. 58) as described previously.

## COMMENTARY

The EMBL‐EBI Job Dispatcher provides a reliable and user‐friendly platform for accessing a wide range of sequence analysis tools through both web interfaces and programmatic interfaces. Built‐in input validation, clear job tracking, and detailed error reporting help users identify common issues, distinguish between input‐ and tool‐related errors, and correct mistakes efficiently. Together with comprehensive documentation and user support, these features help promote efficient and reproducible use of the service, supporting the needs of bioinformatics training and the wider life sciences research community.

### Author Contributions

**Nandana Madhusoodanan**: Conceptualization; writing—original draft; writing—review and editing. **Fábio Madeira**: Conceptualization; writing—original draft; writing—review and editing. **Ania Niewielska**: Project administration; software; writing—review and editing. **Tariq Sulaiman Fazil** Ahamed: Software; writing—review and editing. **Leeroy M. Ncube**: Software; writing—review and editing. **Timothy Porter**: Project administration; resources; supervision; writing—review and editing.

### Conflict of Interest

None of the authors has a conflict of interest to disclose.

## Data Availability

Data sharing is not applicable to this article as no datasets were generated or analyzed.
